# Evaluation of low-pass genome sequencing in polygenic risk score calculation for Parkinson’s disease

**DOI:** 10.1186/s40246-021-00357-w

**Published:** 2021-08-28

**Authors:** Sungjae Kim, Jong-Yeon Shin, Nak-Jung Kwon, Chang-Uk Kim, Changhoon Kim, Chong Sik Lee, Jeong-Sun Seo

**Affiliations:** 1 Precision Medicine Institute, Seoul, 08511 Republic of Korea; 2 Department of Biomedical Sciences, Seoul National University Graduate School, Seoul, 03080 Republic of Korea; 3 Psomagen Inc., Rockville, MD 20850 USA; 4 Department of Neurology, Asan Medical Center, University of Ulsan College of Medicine, 88 Olympic-ro 43-gil, Pungnap 2(i)-dong, Songpa-gu, Seoul, 05505 Republic of Korea; 5 Asian Genome Institute, Seoul National University Bundang Hospital, 172 Dolma-ro, Seongnam, Bundang-gu, Gyeonggi-do 13605 Republic of Korea

## Abstract

**Background:**

Low-pass sequencing (LPS) has been extensively investigated for applicability to various genetic studies due to its advantages over genotype array data including cost-effectiveness. Predicting the risk of complex diseases such as Parkinson’s disease (PD) using polygenic risk score (PRS) based on the genetic variations has shown decent prediction accuracy. Although ultra-LPS has been shown to be effective in PRS calculation, array data has been favored to the majority of PRS analysis, especially for PD.

**Results:**

Using eight high-coverage WGS, we assessed imputation approaches for downsampled LPS data ranging from 0.5 × to 7.0 × . We demonstrated that uncertain genotype calls of LPS diminished imputation accuracy, and an imputation approach using genotype likelihoods was plausible for LPS. Additionally, comparing imputation accuracies between LPS and simulated array illustrated that LPS had higher accuracies particularly at rare frequencies. To evaluate ultra-low coverage data in PRS calculation for PD, we prepared low-coverage WGS and genotype array of 87 PD cases and 101 controls. Genotype imputation of array and downsampled LPS were conducted using a population-specific reference panel, and we calculated risk scores based on the PD-associated SNPs from an East Asian meta-GWAS. The PRS models discriminated cases and controls as previously reported when both LPS and genotype array were used. Also strong correlations in PRS models for PD between LPS and genotype array were discovered.

**Conclusions:**

Overall, this study highlights the potentials of LPS under 1.0 × followed by genotype imputation in PRS calculation and suggests LPS as attractive alternatives to genotype array in the area of precision medicine for PD.

**Supplementary Information:**

The online version contains supplementary material available at 10.1186/s40246-021-00357-w.

## Background

Although the costs of genome sequencing have been reduced over the past decade [1], the expense of whole-genome sequencing (WGS) is still expensive for many genetics studies including genome-wide association studies (GWAS), which require large sample sizes. Instead, genotyping array is preferred in most large-scale studies due to its financial advantages [2]. Low-pass sequencing (LPS) is the type of WGS with genome coverage from 0.5 × to 5.0 × [3, 4]. Since it covers the whole genome with low-coverage, LPS is relatively more cost-efficient compared to deep WGS with coverage around 30 × . Additionally, LPS is advantageous over genotyping arrays in many cases. For instance, genotyping array may have ascertainment bias within assayed SNPs, discovering novel variation both at sample or population level can be feasible when LPS is used [4] so that LPS with genotype imputation increases GWAS power compared to using array [5]. These cases suggested that LPS followed by genotype imputation is a decent alternative to genotyping arrays [6].

Parkinson’s disease (PD) is one of the common neurodegenerative disorders and exerts a significant influence on the world in terms of both healthcare and economy [7]. Although the biggest risk factors of PD include age and numerous environmental factors, several genetic factors also contribute to PD pathogenesis [8]. Understanding the genetic architecture underlying PD is crucial, particularly in developing PD treatments [9]. Despite several GWAS identified PD-associated variants, they poorly explained the observed heritability [10]. Correlations between genetic factors and this disease still remain unclear due to limited understanding of biological functions of causative variants [11] and complex characteristics of PD including heterogeneity and association with multiple genes and pathways [12]. Furthermore, most risk-associated variants for PD were identified from the patients of European ancestry, and little is known for other populations including East Asian populations [11].

Polygenic risk score (PRS) has been widely used for predicting the risk of many complex diseases and traits based on summation of risk alleles and weighted by their effect sizes derived from GWAS results, and it becomes an important factor in the field of precision medicine [13]. Regarding PD, analyzing PRS demonstrated effective predictive power associated with PD symptoms [14, 15]. Here, we performed the PRS analysis to compare predictive power based on genotype array and LPS using risk variants from a meta-GWAS of matched ancestries, to evaluate the efficiency of using LPS for PRS prediction models.

## Results

### Assessing appropriateness of imputation method for low-coverage genotypes

**Fig. 1:**
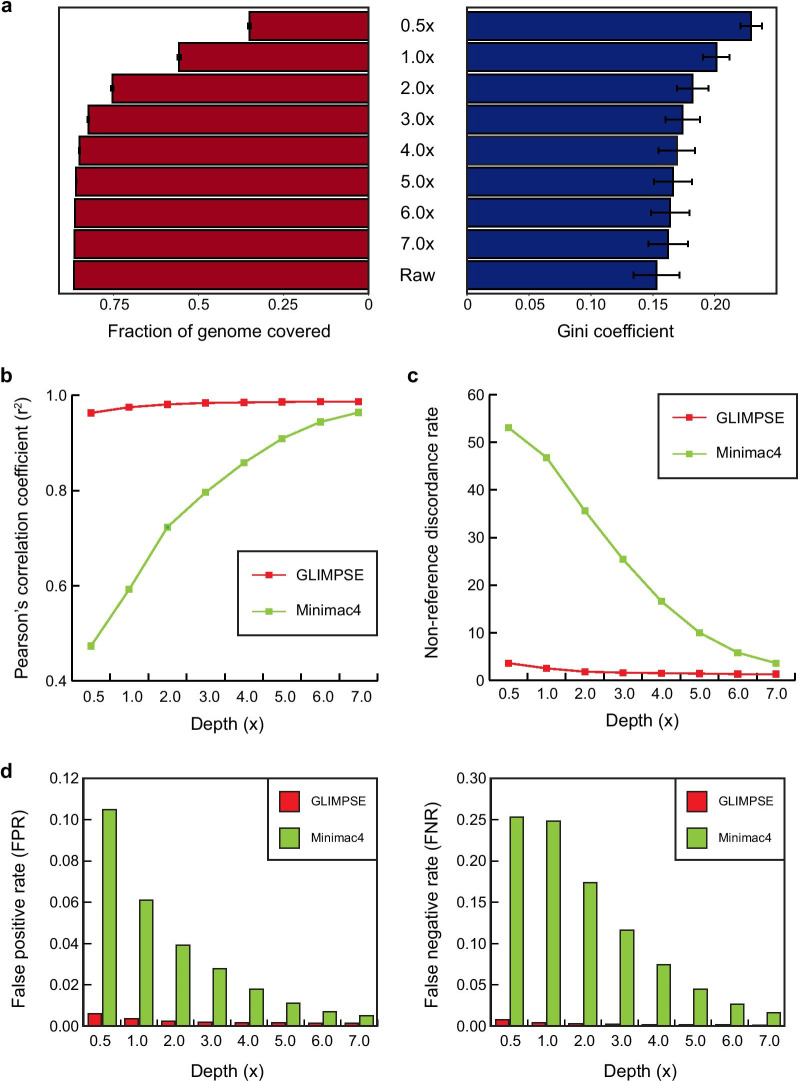
General sequencing statistics and genotype concordance between high-coverage WGS and LPS. **a** Across downsampled LPS and high-depth WGS, left graph shows fraction of whole-genome covered and right shows sequencing read uniformity, measured by Gini coefficients using Lorenz curve constructed with cumulative fraction of sequencing reads within the window size of 10 k base pair of genomic regions. Genotype concordance plots comparing eight high-coverage WGS and LPS constructed by downsampling WGS to low coverage ranging from 0.5 × to 7.0 × . Red and green color represent different imputation approaches; GLIMPSE and Minimac4, respectively. The *x*-axis represents downsampled depth. The *y*-axis represents imputation performances; **b** Pearson’s correlation coefficient (*R*^2^) and **c** Non-reference discordance rate. **d** Details of genotype concordances between high-coverage genotypes and imputed dosages. The rates of false positive (FPR) and negative (FNR) denote mismatches when reference allele in high-coverage but alternates in imputed LPS, and mismatches when alternate allele in high-coverage but reference in imputed LPS, respectively. The *x*-axis represents each LPS depth, and the *y*-axis represents a fraction of each concordance case

The quality of most genotypes from low-coverage is often poor, and sparsely mapped reads likely generate high missing rates of genotypes. Therefore, genotype likelihoods (GL) of low-coverage data need to be updated using the reference panel for more accurate genotype imputation [16, 17]. Recently, the GL imputation and phasing method (GLIMPSE) was developed to iteratively perform haplotype phasing and genotype imputation for LPS data using a Gibbs sampling procedure [16]. To evaluate the validity of this approach for LPS, we compared the imputation accuracy of this method to the traditional imputation using Eagle [18] and Minimac4 [19]. Genotype concordances were measured as Pearson’s correlation coefficients (*R*^2^) and non-reference discordance rates (NDR) between high-coverage and imputed genotypes. We prepared simulated LPS data by downsampling high-coverage WGS to 0.5 × , 1.0 × , 2.0 × , 3.0 × , 4.0 × , 5.0 × , 6.0 × and 7.0 × . Aligned read distribution for each LPS data was presented (Additional file 1: Figure S1). Of the whole genome, the rates of genome covered by sequencing read were 35.0%, 55.7%, 75.3%, 82.4%, 85.0%, 86.0%, 86.4%, 86.5% and 86,6% for 0.5 × , 1.0 × , 2.0 × , 3.0 × , 4.0 × , 5.0 × , 6.0 × , 7.0 × and raw WGS, respectively (Fig. 1a). From 5.0 × LPS, covering rates were increased to the extent of high-depth WGS. We also assessed uniformity of sequencing reads across the genome using the area under Lorenz curve called Gini coefficient. The degree of uniformity can be represented ranging from 0 to 1 where ideal uniformity indicates coefficient of 0 [20, 21]. The average Gini coefficients of LPS were 0.229, 0.201, 0.182, 0.174, 0.169, 0.166, 0.164 and 0.162 for 0.5 × , 1.0 × , 2.0 × , 3.0 × , 4.0 × , 5.0 × , 6.0 × and 7.0 × , respectively, and coefficient of raw high-depth WGS was 0.153. This result demonstrated that distribution of sequencing reads is uniform as sequencing depth increases (Fig. 1a).

As expected, sequencing depth was proportional to *R*^2^ and inversely proportional to NDR when genotype concordances were measured between non-imputed LPS data and high-coverage genotypes (Additional file 2: Figure S2). Then, we compared genotype concordances between high-coverage genotypes and downsampled followed by imputed dosages. Genotype imputation was performed using a merged panel consisting of Northeast Asian Reference Database (NARD) and the 1000 Genomes Project Phase 3 (1KGP3) panel (NARD1) [22]. Consistent with comparison between high-coverage and raw downsampled genotypes, imputed genotypes of higher downsampled depth had improved *R*^2^ with high-coverage genotypes when Eagle and Minimac4 were used for phasing and imputation, respectively. However, GLIMPSE showed constantly high *R*^2^ across downsampled depths (Fig. 1b). The NDR were highly dependent on the depth when Eagle and Minimac4 was used, but GLIMPSE had constantly low NDR across different depths (Fig. 1c). Most importantly, LPS under 3.0 × had very poor imputation accuracy (*R*^2^ = 0.473, 0.592 and 0.723, and NDR = 53.1, 46.8 and 35.6 for 0.5 × , 1.0 × and 2.0 × , respectively) when imputation was conducted using Minimac4. We also calculated genotype concordance using the 1KGP3 panel only, and the results demonstrated a similar pattern with imputed genotypes using NARD1 (Additional file 12: Table S1). Moreover, we identified the rate of false positive (FPR) and false negative (FNR) by comparing high-coverage genotypes and imputed dosages using two different methods. Using GLIMPSE had constantly low FPR and FNR which were below 1% across low depths while the other approach had high FPR and FNR, especially below 3.0 × (FPR: 10.5%, 6.10%, 3.93% and 2.77%, FNR: 25.3%, 24.8%, 17.3% and 11.6% at 0.5 × , 1.0 × , 2.0 ×  and 3.0 ×, respectively; Fig. 1d).

### Imputation performance of LPS and array at different allele frequencies

**Fig. 2:**
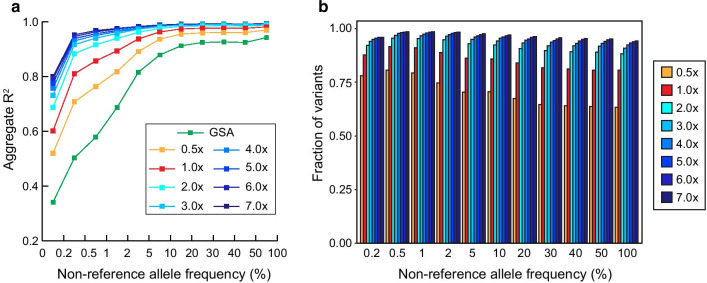
Imputation accuracy comparison across allele frequency bins. **a** Imputation accuracy of simulated GSA and downsampled LPS constructed by 8 WGS at each frequency bin. Two different approaches; GLIMPSE and Minimac4 were used for imputing downsampled LPS and simulated GSA, respectively. The *x*-axis represents non-reference allele frequency (AF) of East Asian population derived from the gnomAD v3.1. Variants were defined to be rare, low and common when AF < 0.5%, 0.5% ≤ AF < 5% and AF ≥ 5%, respectively. The *y*-axis represents aggregate *R*^2^ of variants between high-coverage genotypes and imputed dosages at each frequency bin. **b** Direct comparison of imputation accuracy using overlapping imputed SNPs with *R*^2^ > 0.8 between GSA and downsampled LPS. The *x*-axis represents non-reference allele frequency (AF) of East Asian population derived from the gnomAD v3.1. The *y*-axis represents the fraction of SNPs that were more accurately imputed SNPs in downsampled LPS

We primarily compared the average number of typed and imputed variants across eight individuals. We observed relatively more typed variants at 0.5 × than simulated array (670 k vs 1.66 million SNPs for array and 0.5 × LPS, respectively; Additional file 3: Figure S3). Also, larger portion of typed variants by increasing sequencing depths, and plateau from 5.0 × (Additional file 3: Figure S3). To further investigate imputation performance for LPS using GLIMPSE, we compared imputation accuracy of 4,958,741 overlapping SNPs across LPS data at different allele frequencies (AF). Along with LPS, we created a simulated genotype array data by extracting genotypes at global screening array (GSA) regions from high-coverage WGS to compare performance between array and LPS. Non-reference AF bins were determined based on AF of East Asian from the Genome Aggregation database (gnomAD) v3.1 database [23]. Consistent with the previous results [16, 24], we discovered that imputed dosages from LPS were relatively more accurate than those from GSA at each AF, particularly at rare AFs. Within LPS data, the depth and overall imputation accuracy of each LPS were proportional as expected. For rare (< 0.5%) variants, all of LPS data had *R*^2^ of below 0.8, especially, 0.5 × had deficient results (aggregate *R*^2^ = 0.52 and SD = 0.46, and aggregate *R*^2^ = 0.71 and SD = 0.39 for AF < 0.2% and 0.2% ≤ AF < 0.5%, respectively; Fig. 2 and Additional file 12: Table S2). The *R*^2^ of ultra-low coverage (< 3.0 ×) at rare and low frequency (AF < 5%) were lower than those of LPS with > 3.0 × . The differences in imputation accuracy between each LPS were diminished as AF increases (from AF > 5%), and subtle differences were observed for SNPs with AF > 50% (Fig. 2a). Generally, SNPs with imputation scores (*R*^2^) of > 0.8 were used in GWAS [12], and considered to be high quality. Consistent with Fig. 2a, the number of accurately imputed SNPs was relatively higher in LPS than GSA at each non-reference allele frequency bin, particularly differences in quality were higher at rare frequency bins (Additional file 4: Figure S4). As the *R*^2^ of variants at rare frequency bins were highly variable (Additional file 12: Table S2), we directly compared the fraction of high imputed quality SNPs (*R*^2^ > 0.8) that were more accurately imputed among the overlapping imputed SNPs between GSA and LPS. The quality of most SNPs (> 0.6) were more decent in LPS, and from 3.0 × , more than 90% of SNPs were more accurate across every allele frequency bins. Also, the fractions in these LPS were diminished as allele frequencies are increasing at ultra-low coverage (0.5 × and 1.0 ×) (Fig. 2b).

### Selecting PD-associated SNPs for PRS calculation

The GWAS summary statistics of 23 million SNPs for PD from the UK Biobank study of 1239 PD cases and 451,025 controls of European ancestry (UK Biobank G20) [25], and 74 SNPs that were previously identified to be associated with PD from several GWAS using European populations (EUR total) [12]. However, the GWAS results from a certain population need to be carefully selected and used for predicting disease risks [26]. To avoid unwanted bias arising from unmatched populations between GWAS results and target data, we additionally selected PD-associated risk SNPs derived from a large-scale meta-GWAS of 6,724 PD cases and 24,851 controls from East Asian populations [12] (Table 1). We prepared three identified SNP sets for East Asians to construct PRS model from this GWAS: (1) the 11 East Asian SNPs that were defined as genome-widely significant (EAS; threshold: *P* < 5.00 × 10^–8^) in a meta-GWAS, (2) the 9 previously identified to be associated with PD in European populations that were replicated in this meta-GWAS (EUR replicated; threshold: *P* < 1.00 × 10^–5^) and (3) combined EAS and EUR replicated SNPs (Combined set). For the Combined set, we excluded less significant SNPs within the same linkage disequilibrium blocks (*R*^2^ ≥ 0.5) using LDmatrix [27]. As previously stated in a meta-GWAS, most PD-associated SNPs were common SNPs (effect AF > 5%) from gnomAD v3.1, but only rs141336855 had AF of 0.1% from gnomAD v3.1 global, but 2.6% and 2.4% from gnomAD v3.1 East Asian and NARD, respectively. Also the effect AF of rs16846351 was 1.6% at global population, but AF > 5% for East Asian population. Among these PD-associated SNPs, the 2 SNPs were typed in GSA data, and others were imputed. The missing rates and average depths per each SNP were consistent with their downsampled coverage (Additional file 12: Table S3). We further tested imputation accuracy of selected PD-associated SNPs using 8 high-coverage WGS, and high imputation accuracy for 0.5 × , 1.0 × and 2.0 × data were achieved when GLIMPSE was used (average *R*^2^ = 0.998 and 0.992 for NARD1 and 1KGP3 panel, respectively; Additional file 5: Figure S5 and Additional file 12: Table S4).

**Table 1:** Parkinson's disease associated risk SNPs from an East Asian meta-genome-wide association study (Foo et al. [20])

rs ID	Chromosome	Position	Effect allele	Feature^a^	Effect allele frequency			
					gnomAD v3.1 ALL	gnomAD v3.1 EAS	NARD ALL	NARD KOR
rs823118	1	205,723,572	T	EUR replicated	45.7%	54.3%	51.4%	52.5%
rs6679073	1	205,756,484	A	EAS	22.0%	53.3%	49.7%	51.2%
rs16846351	1	226,846,712	G	EAS	1.6%	6.3%	6.0%	6.4%
rs4653767	1	226,916,078	T	EUR replicated	72.7%	72.1%	23.8%	22.2%
rs2292056	3	182,735,211	T	EAS	77.9%	41.5%	59.9%	63.1%
rs12637471	3	182,762,437	G	EUR replicated	75.3%	42.0%	59.9%	62.9%
rs34311866	4	951,947	C	EUR replicated	14.2%	13.8%	17.7%	16.9%
rs11724635	4	15,737,101	A	EUR replicated	43.5%	37.5%	36.9%	34.3%
rs3816248	4	77,101,068	T	EAS	86.6%	66.1%	32.5%	33.4%
rs356182	4	90,626,111	G	EUR replicated	35.2%	66.2%	31.6%	29.1%
rs6826785	4	90,682,474	C	EAS	21.7%	54.8%	54.7%	55.5%
rs246814	5	75,599,208	T	EAS	9.6%	9.1%	NA	NA
rs1887316	6	112,151,452	G	EAS	81.1%	87.9%	12.4%	14.2%
rs997368	6	112,243,291	A	EUR replicated	65.5%	64.2%	39.3%	39.6%
rs9638616	7	70,750,493	T	EAS	37.9%	49.2%	55.0%	55.1%
rs12278023	11	83,510,117	T	EAS	55.1%	50.2%	47.7%	48.6%
rs3793947	11	83,544,472	G	EUR replicated	57.4%	53.4%	46.3%	47.0%
rs141336855	12	40,387,749	T	EAS	0.1%	2.6%	2.4%	2.4%
rs12456492	18	40,673,380	G	EUR replicated	32.8%	38.0%	42.7%	40.9%
rs4130047	18	40,678,235	C	EAS	32.2%	37.8%	42.6%	40.7%

^a^Feature: EAS represents significant SNPs from this GWAS (*P* < 5.00 × 10–8); EUR replicated represents replicated SNPs in this GWAS (*P* < 1.00 × 10–5)

### Comparative PRS analysis between genotyping array and LPS for PD

We prepared GSA and WGS with an average depth of 5.0 × data of 188 individuals to perform comparative PRS analysis for PD risk prediction. To evaluate the efficiency of ultra-low coverage, we downsampled WGS to 0.5 × , 1.0 × and 2.0 × (Additional file 6: Figure S6). Currently, pruning and thresholding (P + T) method is one of the most widely used calculation approach to construct PRS model with the LD and *P*-value as parameters. In addition to P + T approach, several Bayesian approaches for PRS calculation have been continuously developed. We therefore used P + T method and Bayesian approaches; PRScs [28] and EB-PRS [29] with and without reference LD information, respectively. Using these multiple approach for PRS calculation, we assessed the discrimination of PRS between PD cases and controls using area under curve (AUC) metrics. To extensively evaluate the performances in PRS, we first calculated PRS based on SNP sets derived from the UK Biobank study [25] and 74 known SNPs from European populations [12] to leverage larger number of SNPs regardless of matched ethnicity. Then, we conducted unadjusted PRS analysis using a total of three different combinations of significantly associated SNPs with PD in East Asian populations as described in the previous section. For P + T, the best AUC was chosen among the multiple *P* value thresholds (Additional file 7: Figure S7).

**Fig. 3:**
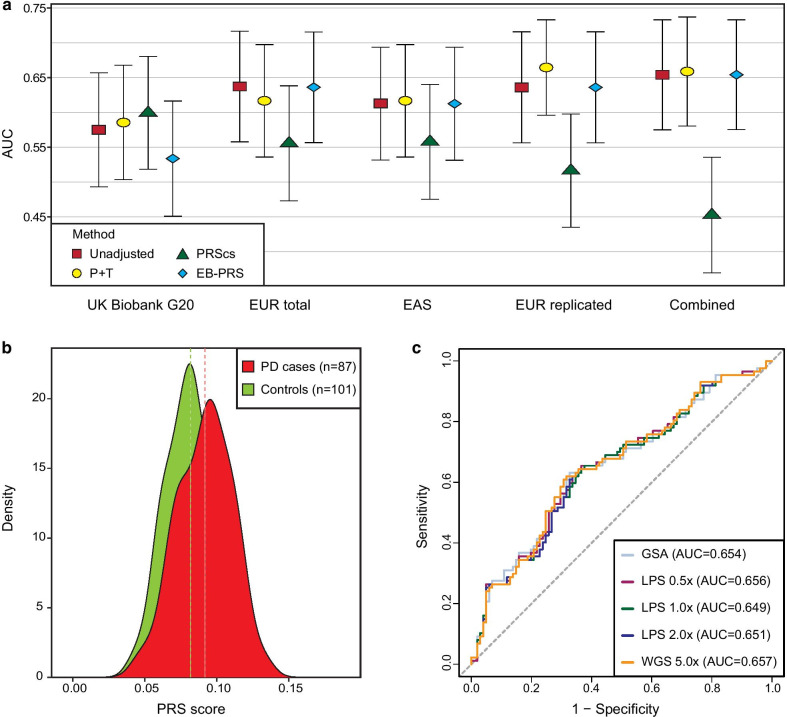
Assessment of PRS models based on SNP sets from GWAS in European and East Asian populations. **a** Evaluation of 5 different PRS models using 4 different PRS approaches based on data of GSA followed by imputation. The *x*-axis presents PRS models: UK Biobank G20; GWAS summary statistics of PD from the UK Biobank study of European populations, EUR total; 74 previously identified PD-associated SNPs in European populations, EAS; 11 genome-wide significant SNPs in a meta-GWAS of East Asians (*P* < 5.00 × 10^–8^), EUR replicated; 9 SNPs in EUR total that were replicated in a meta-GWAS (*P* < 1.00 × 10^–5^), and Combined; 16 SNPs of EAS and EUR replicated that were LD clumped. A total of 4 different approaches for PRS calculations were used: unadjusted, P + T, PRScs, and EB-PRS. The area under curve (AUC) with 95% confidence intervals are shown in the *y*-axis. **b** Score distributions of 87 PD cases and 101 controls using GSA based on the Combined set. Red color and green color represent PD cases and controls, respectively. Mean score values of each group were presented as dashed lines. **c** AUC of the Combined set using GSA, low-coverage WGS (5.0 × average) and downsampled LPS 0.5 × , 1.0 × and 2.0 ×

The AUC using UK Biobank G20 and EUR total were approximately from 0.50 to 0.60 and the average AUC of four different approaches based on the Combined set were 0.605 which was the highest AUC among the five different PRS sets (Fig. 3a). Using SNP sets from the East Asian study, we observed dramatic drop in AUC when PRScs was used. And we found that only 27.0%, 45.5%, 22.2% and 37.5% of whole EUR total, EAS, EUR replicated and Combined, respectively, were considered for PRS calculation using PRScs. Also, only single SNP in the EUR replicated set was taken into account for P + T approach. In addition to AUC results using GSA, the patterns of AUC using LPS and raw WGS (5.0 ×) were highly homogeneous with those using GSA and slight improvement for PRScs when LPS were used (Additional file 8: Figure S8). Overall, both GSA and low-coverage WGS showed that using SNPs that were significant in East Asians had relatively higher AUC.

Using significantly PD-associated SNPs in East Asian population, density plots of PRS using GSA data demonstrated that the distribution of standardized PRS for PD cases were shifted to the right compared to those of controls, and mean score of 0.0916 vs 0.0816 for PD cases and controls, respectively (Fig. 3b). Besides, we observed that PRS using LPS had comparable shifting pattern with GSA, indicating that higher PRS within PD cases than controls (Additional file 9: Figure S9). We illustrated the receiver operating characteristic (ROC) curves of East Asian sets, the unadjusted AUC of a PRS model based on the Combined set had an average of 0.653 (0.654; 95% CI 0.575–0.733, 0.656; 95% CI 0.577–0.734, 0.649; 95% CI 0.570–0.728, 0.651; 95% CI 0.572–0.730 and 0.657; 95% CI 0.578–0.735 for GSA, LPS 0.5 × , LPS 1.0 × , LPS 2.0 × and WGS 5.0 × , respectively; Fig. 3c). We compared the predictive power of PRS using different types of genomic data; GSA and LPS, and there was no significant difference in scores between GSA and LPS 0.5 × , 1.0 × 2.0 × and WGS 5.0 × on same PRS models (*P* > 0.5, *F* value = 0.018, 0.008 and 0.004 for EAS, EUR replicated and Combined SNPs, respectively; Fig. 3c). Also we found negligible differences in AUC between GSA and low coverage WGS data when PRS were computed using the EAS and EUR replicated which had average AUCs of 0.614 and 0.638, respectively (Additional file 10: Figure S10). The PRS calculated by imputed genotypes using the 1KGP3 panel had average AUC of 0.616, 0.627 and 0.650 for EAS, EUR replicated and Combined set, respectively (Additional file 12: Table S5). Our results demonstrated successful replication of PD SNPs in our cohort and were consistent with a meta-GWAS result showing improvement in AUC when two SNP sets were combined [12].

**Fig. 4:**
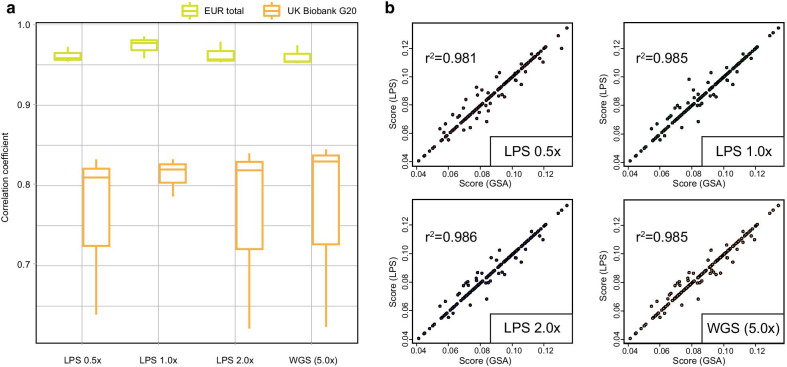
Correlation of PRS calculated using genotype array and low-pass sequencing data. Correlation plots between calculated PRS using LPS and GSA of 188 individuals. **a** Correlation coefficient between low-coverage WGS and array using unadjusted, P + T, PRScs and EB-PRS for EUR total and UK Biobank G20 set. **b** PRS were calculated based on 16 PD-associated SNPs from an East Asian meta-GWAS. Correlation coefficients between scores from GSA and LPS were presented. The *x*-axis represents the scores using GSA, and the *y*-axis represents the scores using LPS; 0.5 × , 1.0 × , 2.0 × and raw WGS (5.0 × average)

Furthermore, strong correlations were discovered between calculated scores using GSA and different coverage of LPS. The mean correlation coefficients were above 0.95 and 0.80 when EUR total and UK Biobank G20 were used, respectively (Fig. 4a), but we observed relatively lower correlation coefficient between GSA and LPS for UK Biobank G20 using P + T approach due to larger differences in the number of SNP between GSA and LPS. For scores based on the Combined set using East Asian SNPs, correlation coefficients were > 0.98 for all LPS data (0.981, 0.985, 0.986 and 0.985 for 0.5 × , 1.0 × , 2.0 × and WGS 5.0 × ; Fig. 4b). Other PRS sets using significant SNPs in the East Asian study (EAS and EUR replicated) also had robust correlation between GSA and LPS data (*R*^2^ > 0.98; Additional file 11: Figure S11), and *R*^2^ > 0.95 when the 1KGP3 reference panel was used for imputation (Additional file 12: Table S6).

## Discussion

Previously, genotype concordances between low-coverage (~ 0.5 ×) and genotype array, and deep sequencing data (~ 30 ×) were highly correlated, and several studies have continuously demonstrated potentials of LPS for precision medicine [4, 6]. Additionally, LPS under 1.0 × has shown strong advantages over genotype array in terms of cost and imputation accuracy [24, 30]. Typically, LPS around 1.0 × is expected to be half of the cost of genotype array with less than 1 million variants. With a high-quality imputation derived from a decent reference panel, LPS under 1.0 × can be more suitable for large-scale genetic studies. To assess availability of LPS, we first evaluated the effect of newly established imputation for LPS using various low-coverage depths. High coverage WGS were downsampled ranging from 0.5 × to 7.0 × , and we observed that fixed genotype calls of downsampled WGS were highly incorrect compared to high-coverage genotypes (Additional file 2: Figure S2). This inaccuracy of genotype call is the culprit for extremely low imputation accuracy using a traditional approach implemented hidden Markov model which takes diploid genotypes of target samples into account to compute probability of diploid [18]. Inferred haplotype pairs based on unreliable genotype calls would result inaccurate filling the gaps between the markers. Therefore, probabilistic form of genotypes called GL should be used for low-coverage data instead of genotype calls to consider all possible genotype possibilities based on mapping and quality scores [16], and imputation which updates GL would resolve unreliability of imputed LPS and showed high imputation accuracy. Although refining GL requires high computational burden, GLIMPSE shows fast and accurate imputation calls using a novel linear time sampling algorithm which is appropriate for the size of large reference panel [16]. This result validated that appropriate imputation for LPS is crucial, particularly for obtaining correct alternate alleles (Fig. 1).

Further assessment on imputation accuracy presented that LPS for rare (< 0.5%) and low (< 5%) variants still had relatively lower accuracies than common variants, but outperformed when simulated GSA data was imputed. The aggregate *R*^2^ and direct comparison of overlapping SNPs between LPS and GSA demonstrated that higher imputation accuracies were obtained from LPS data, especially at rare frequency bins (Fig. 2a). This results suggest that LPS would be beneficial for rare variant imputation. We demonstrated that sequencing reads are sparsely covered more than half of the entire genome with decent uniformity at low coverage from 1.0 × (Fig. 1a). Since dense genotype array only covers relatively small amount of genome, more accurate imputed genotypes can be potentially obtained by leveraging more number of GL from sequencing reads than a traditional approach using genotype array. One of the strategies to overcome this missing heritability of PD is capturing rare variants by increasing sample size of the studies or covering the whole genome including non-coding regions for identifying more promising candidates [10]. Our results suggest that in the case of when rare and pathogenic variants were prevalent in disease-cases with low PRS [31], LPS followed by imputation would increase the power of PRS by combining accurately imputed rare pathogenic variants using the population-specific reference panel.

We selected a cohort of Parkinson’s disease (PD) because it is one of the most common neurodegenerative diseases with complex genetic characteristics. Even though substantial efforts have been devoted to elucidate the complex genetic architecture of PD, predicting early diagnosis of PD is still challenging due to missing heritability of this disease. To evaluate the performance of LPS for predicting a complex disease, we prepared a cohort of 188 Korean individuals including 87 PD cases and 101 controls and generated genotyping array and WGS data to an average depth of 5.0 × , which is known to be the minimum coverage for accurately detecting genome variation [32], and showed high genome concordance with 30 × [3]. Primarily, the PRS analyses were performed by leveraging genome-wide SNPs and calculation methods including P + T and Bayesian models with and without reference LD information. In terms of predictive power of PRS, both LPS and GSA showed poor performances overall, potentially due to utilizing SNPs from European populations. The results of AUC using multiple PRS calculation approach with different sets of SNPs highlighted that PRS based on matched population is important for more accurate PRS analysis. Selecting PD-associated SNPs as base data for PRS calculation should be carefully addressed because risk AF and PRS were inconsistent across the ethnicity groups, and such bias could cause misestimation of genetic disease risks [24, 26]. Since the majority of GWAS for a variety of traits and diseases including PD are biased to European ancestries [11], we therefore chose significantly identified PD-associated SNPs from a meta-GWAS consisting of East Asian individuals for further analysis. The performances of PRS were highly variable across tools and sets as significantly identified SNPs for PD in the East Asian meta-GWAS were less than 20, and PRScs limitedly utilized SNPs due to reference LD information (Fig. 3a).

Whether using genome-wide SNPs around 100 k or population-specific SNPs, correlation of individual scores between LPS and array were generally high, particularly for East Asian SNPs (Fig. 4). Along with higher accuracy for rare variant compared to the array, this homogeneity of common SNPs between array and LPS suggest that LPS would be valuable in PRS analysis. Regarding the power of PRS, although our result showed below the level of accurate prediction, a number of PRS models had been demonstrated that PRS with clinical information would increase PD predictions [33, 34]. Additionally, we expected that mapping PRS using LPS with data from emerging technologies such as machine learning and single-cell RNA sequencing would improve the power of prediction and elucidate the genetics of PD [11].

## Conclusion

We demonstrated the potential of LPS with coverage less than 1.0 × to be used for predicting PD, and suggested cost-efficient LPS to replace GSA data which have been widely and popularly used in this field. Therefore, we believe that utilization of LPS could become useful in precision medicine with financial and technical advantages over genotype array.

## Materials and methods

### Data collection and sequencing

A total of 188 Korean individuals, consisting of 87 individuals diagnosed with PD and 101 controls, were collected at Asan Medical Center (Seoul, South Korea). High-depth WGS were generated using additional eight Koreans without PD diagnosis from the cohort for evaluating imputation performance of downsampled data. Genomic DNA from the blood of collected individuals were extracted and prepared. All quality control passed blood genomic DNA samples were subjected to library preparation with the Illumina Nextera DNA Flex kit (Illumina, USA) following manufacturer’s instruction. Briefly, input genomic DNA was treated with bead-linked transposomes. After tagmentation stop reaction and purification, tagmented genomic DNA was amplified by PCR reaction with recommended cycles described in the manufacturer's instruction. Library was quantified both with the quantitative PCR method (KAPA Library Quantification Kit; Kapa Biosystems, USA) and fluorescent method (Qubit dsDNA HS assay Kit; Thermo Fisher Scientific, USA). Each constructed and measured library was normalized by diluting with the calculated amount of nuclease-free water, and all normalized libraries were pooled and then sequenced with the Illumina NovaSeq 6000 platform (Illumina, USA) based on the manufacturer’s instruction.

### Data processing

Obtained DNA was genotyped on the customized Global Screening Array (GSA; Illumina, USA) which captured multiethnic genetic variation. Genotypes were processed to variant call format (VCF) files according to the manufacturer’s guide using Illumina’s GenomeStudio and the in-house processing method. Produced individual VCFs were called and merged using GATK v4.1.2 [35], then variants were normalized using bcftools v1.3.1 [36]. Raw sequencing data was processed based on the GATK’s best practice with the following steps: Sequence trimming using Trimmomatic, read alignment to the human reference genome (hg19) using BWA v0.7.17 [37], sort BAM file and mark duplicate reads using Picard v2.18.25, and base recalibration and haplotype call were conducted using GATK v4.1.2.

### Downsampling and coverage distribution

To evaluate the efficiency of LPS coverage, we downsampled eight high-coverage (average depth of 27.2 ×) WGS to 0.5, 1.0, 2.0, 3.0, 4.0, 5.0, 6.0 and 7.0 × using SAMtools view [36] based on the calculated proportion for subsampling WGS to low-coverage data. For PRS analysis using 188 individuals including the PD cases and controls, we also downsampled raw sequencing data to 0.5, 1.0 and 2.0 × . Coverage distributions for downsampled data were calculated using aligned read count per genotypes. The Gini coefficients were calculated using the ratio of area under the Lorenz curve which was generated by cumulative fraction of sequencing reads and genomic regions. We set the size of window for calculating number of reads as 10 k base pair length.

### Phasing and imputation

We performed genotype phasing using Eagle v2.4 [18] and imputation using Minimac4 [19] based on the 1KGP3 [38] which is the most conventional panel and an East Asian specific reference panel, called the NARD [22] merged with 1KGP3. After imputation, we filtered variants with information score below 0.3, and remaining imputed genotypes were converted into PLINK [39] binary format for further analyses. Also we conducted genotype imputation for LPS data using GLIMPSE. Mapped reads at only bi-allelic sites of each reference panel were extracted from LPS BAM data using bcftools mpileup because the presence of indels might affect the imputation quality [16]. Then iterative refinement of GL using the reference panels with segmentation size of 2 Mb with buffer size of 200 kb produced imputed dosages and multiple chunks within each chromosome were ligated.

### Genotype concordance assessment

We compared raw and imputed downsampled to high-coverage WGS to assess genotype concordance for evaluating imputation performance. We extracted overlapping variants between two sets of WGS. Pearson’s correlation coefficient (*R*^2^) and non-reference discordance rates were computed using bcftools stats. We extracted a total of 1,373,903 overlapping variants between different depths of non-imputed downsampled LPS and high-coverage WGS, to compare genotype concordances. For assessing two different imputation approaches, 5,371,175, 5,465,923, 5,468,916, 5,469,108, 5,469,158, 5,469,208 and 5,469,213 overlapping variants between approaches were used for 0.5 × , 1.0 × , 2.0 × , 3.0 × , 4.0 × , 5.0 × , 6.0 × and 7.0 × , respectively.

### PRS calculation

Calculating PRS requires two types of data; GWAS summary statistics including known risk allele with their effect sizes are called base data, and individual-level genotype data with their phenotypes are called target data [40]. We converted bi-allelic genotyped and imputed autosomal SNPs of each GSA and LPS into PLINK2 binary format. For P + T approach, we conducted LD clump using PLINK [39] with a LD parameter of 0.5 and *P* value thresholds were set ranging from 5.00E-02 to 1.00E-20. Bayesian approaches including PRScs [28] and EB-PRS [29] were conducted with default parameters. For PRScs, we used reference LD information of 1KGP3 for East Asian populations. Summation of the number of risk alleles weighted by their effect size from an East Asian meta-GWAS summary statistic [12]. Individual scores were calculated as below:

$$\mathrm{PRS} = \sum_{k=1}^{k}{w}_{k}{X}_{k}$$

where *k* is PD-associated SNP, *w* is the effect size as weight and *X* is the number of effect alleles (risk alleles). Calculated scores were normalized to have mean zero using PLINK. Area under the curve (AUC) of PRS for each variant set was estimated using scikit-learn libraries [41].

## Supplementary Information

**Additional file 1: Figure S1.** Distribution of aligned read per genotype of downsampled WGS of eight individuals. The *x*-axis represents the number of read counts aligned to the genotypes. The *y*-axis represents a fraction of genotypes from downsampled WGS of eight individuals.

**Additional file 2: Figure S2.** Genotype concordance between high-coverage genotypes and non-imputed genotypes. The *x*-axis represents each downsampled depths, and the *y*-axis represents **a,** Pearson’s correlation coefficient (*R*^2^), and **b,** Non-reference discordance rate.

**Additional file 3: Figure S3.** Number of typed and imputed variants in million from simulated array (GSA) and downsampled LPS from 0.5 × to 7.0 × .

**Additional file 4: Figure S4.** Number of variant with *R*^2^ > 0.8 from simulated array (GSA) and downsampled LPS from 0.5 × to 7.0 × across each frequency bins. The *x*-axis represents non-reference allele frequency (AF) of East Asian population derived from the gnomAD v3.1. The *y*-axis is the number of variants in log scale.

**Additional file 5: Figure S5.** Comparison of imputation approach using 20 PD-associated SNPs. Red indicates imputation using GLIMPSE, and green indicates haplotype phasing using Eagle v2.4 and Minimac4 for imputation. **a,** Pearson’s correlation coefficient (*R*^2^) and **b,** Non-reference discordance rate.

**Additional file 6: Figure S6.** Distribution of aligned read per genotype of downsampled WGS of 188 individuals. The *x*-axis represents the number of read counts aligned to the genotypes. The *y*-axis represents a fraction of genotypes from downsampled WGS of 188 individuals.

**Additional file 7: Figure S7.** AUC of 5 different PRS sets at each *P* value threshold. After LD clump, multiple *P* value thresholds were set to assess AUC values. The *x*-axis represents significance thresholds from 5.00 × 10^–2^ to 1.00 × 10^–20^, and the *y*-axis represent AUC.

**Additional file 8: Figure S8.** Assessment of PRS models based on SNP sets from GWAS in European and East Asian populations using LPS. Evaluation of 5 different PRS models using 4 different PRS approaches based on data of GSA followed by imputation. The *x*-axis presents PRS models: UK Biobank G20; GWAS summary statistics of PD from the UK Biobank study of European populations, EUR total; 74 previously identified PD-associated SNPs in European populations, EAS; 11 genome-wide significant SNPs in a meta-GWAS of East Asians (*P* < 5.00 × 10^–8^), EUR replicated; 9 SNPs in EUR total that were replicated in a meta-GWAS (*P* < 1.00 × 10^–5^), and Combined; 16 SNPs of EAS and EUR replicated that were LD clumped. A total of 4 different approaches for PRS calculations were used: unadjusted, P + T, PRScs, and EB-PRS. The area under curve (AUC) with 95% confidence intervals is shown in the *y*-axis.

**Additional file 9: Figure S9.** Density plots using 16 PD-associated SNPs from LPS data. Green color represents density for cases, and pink color represents for control. The *x*-axis represents polygenic risk score, and the *y*-axis represents density of samples.

**Additional file 10: Figure S10.** AUC of PRS analysis. Genotype imputation was conducted using the NARD reference panel by GLIMPSE. **a** PRS calculated based on 11 Asian SNPs, **b** PRS calculated based on nine European SNPs that were replicated in East Asian cohorts.

**Additional file 11: Figure S11.** Correlation of PRS between GSA and LPS. **a** using 11 Asian SNPs, **b** using nine European SNPs that were replicated in East Asian cohorts.

**Additional file 12:**  Supplementary Tables.

## Data Availability

Please contact author for data requests.

## Abbreviations

PD: Parkinson’s disease
PRS: Polygenic risk score
LPS: Low-pass sequencing
GSA: Global screening array
GL: Genotype likelihoods
NARD: Northeast Asian Reference Database
GWAS: Genome-wide association studies
WGS: Whole-genome sequencing
SNP: Single-nucleotide polymorphism
VCF: Variant call format
*R*^2^: Correlation coefficient
SD: Standard deviation
