# Supplementary figures and images for: Evaluation of low-pass genome sequencing in polygenic risk score calculation for Parkinson’s disease
Source: Hum Genomics. 2021 Aug 28;15:58. doi: 10.1186/s40246-021-00357-w (PMC8403377; doi:10.1186/s40246-021-00357-w)

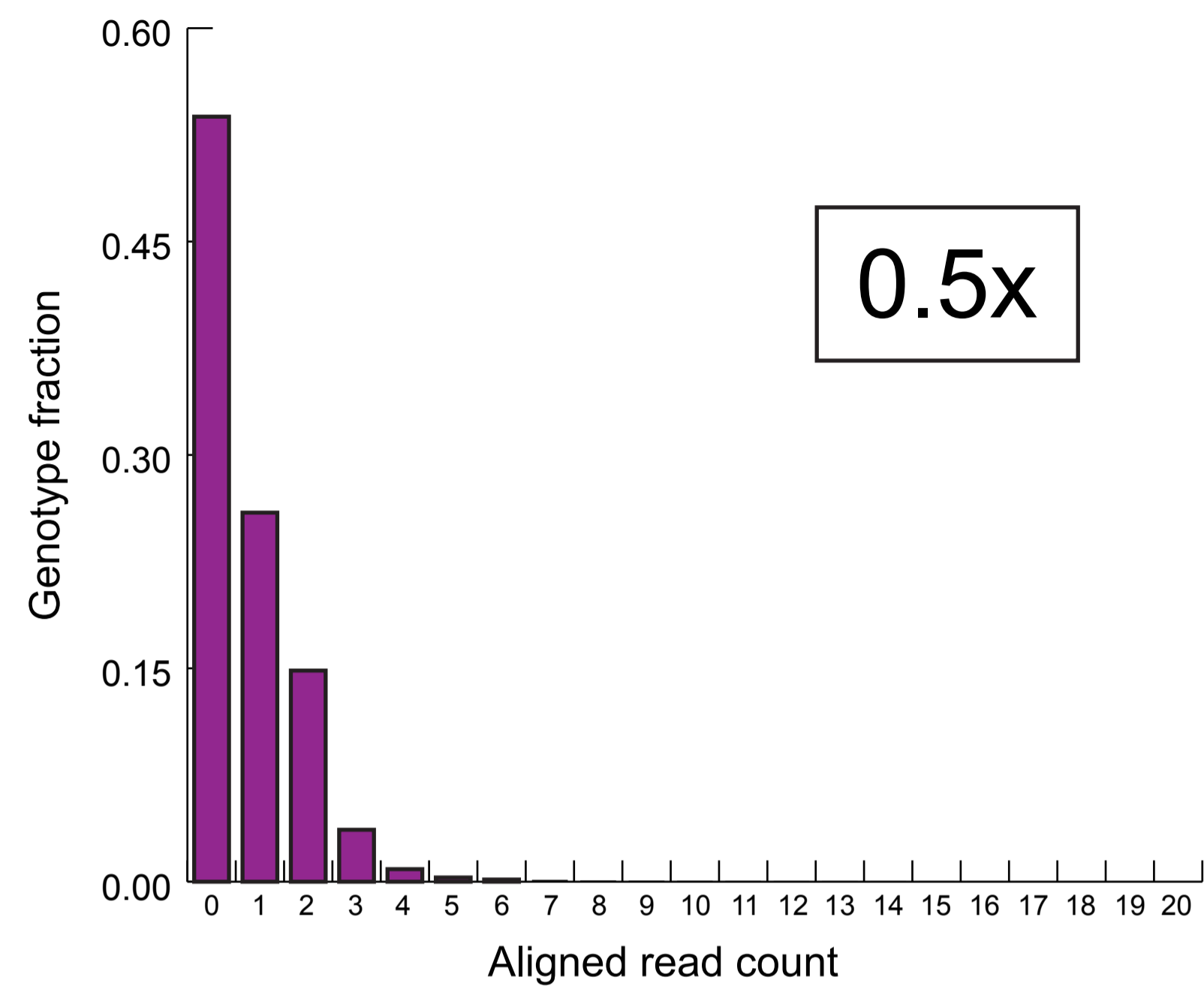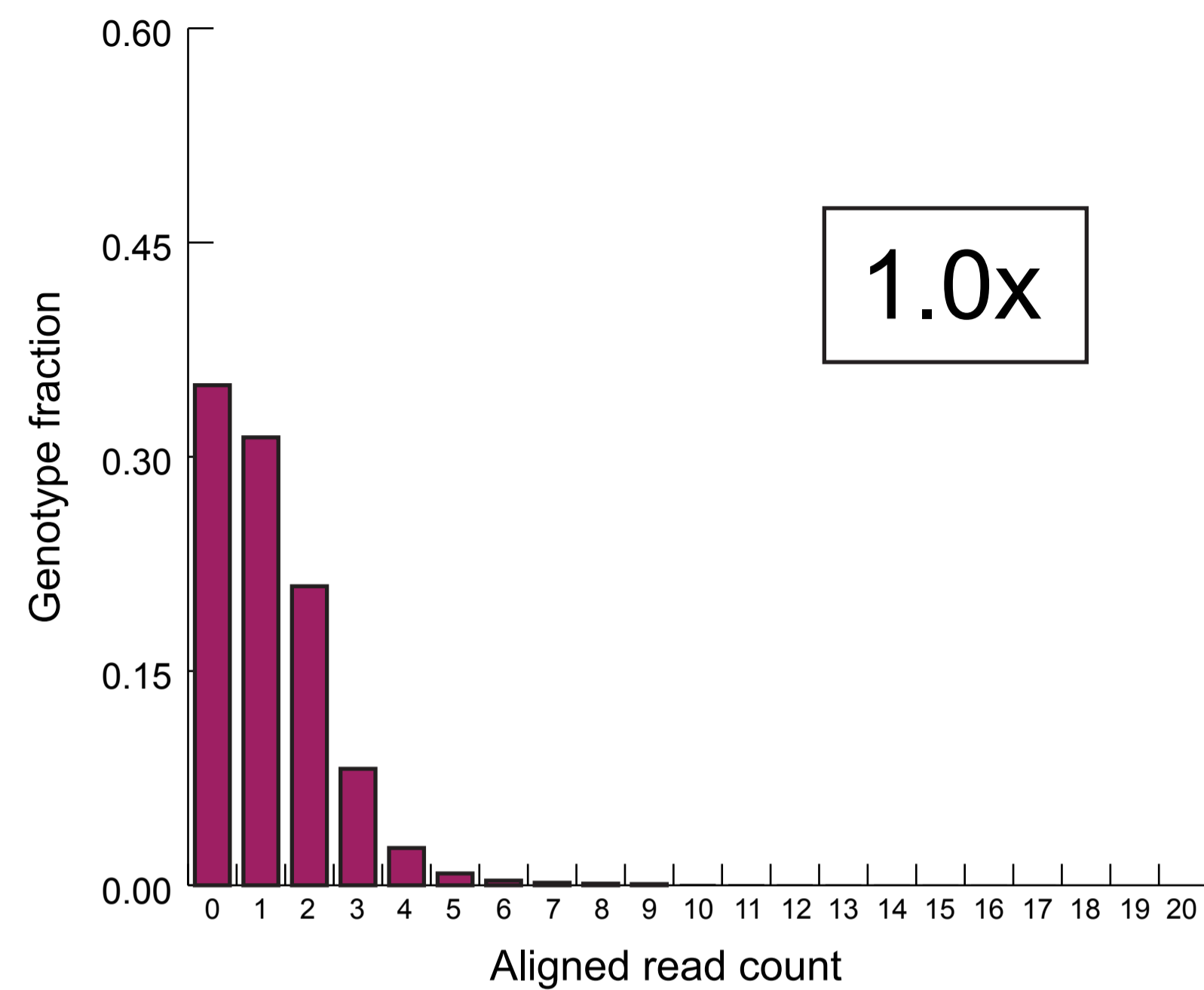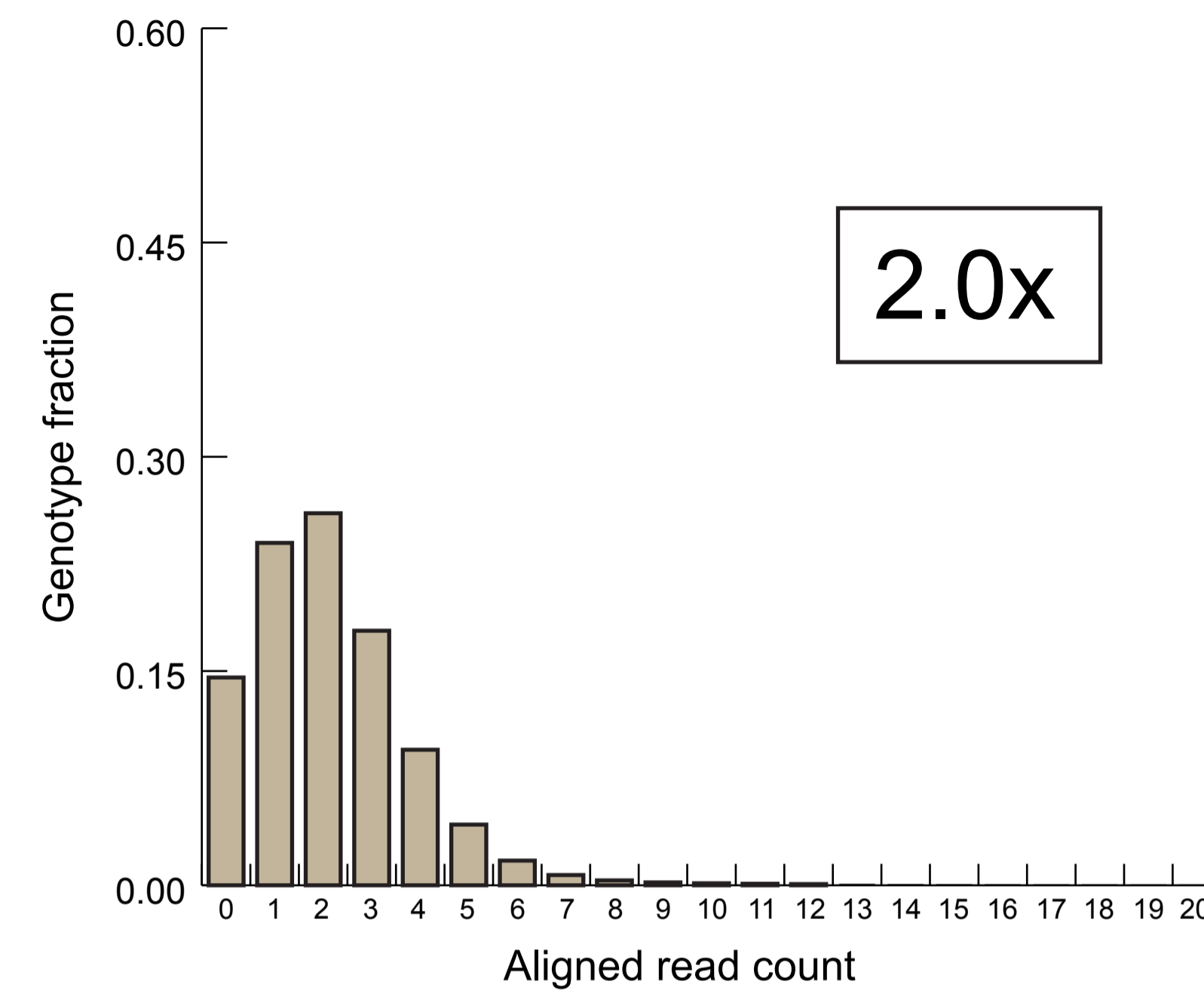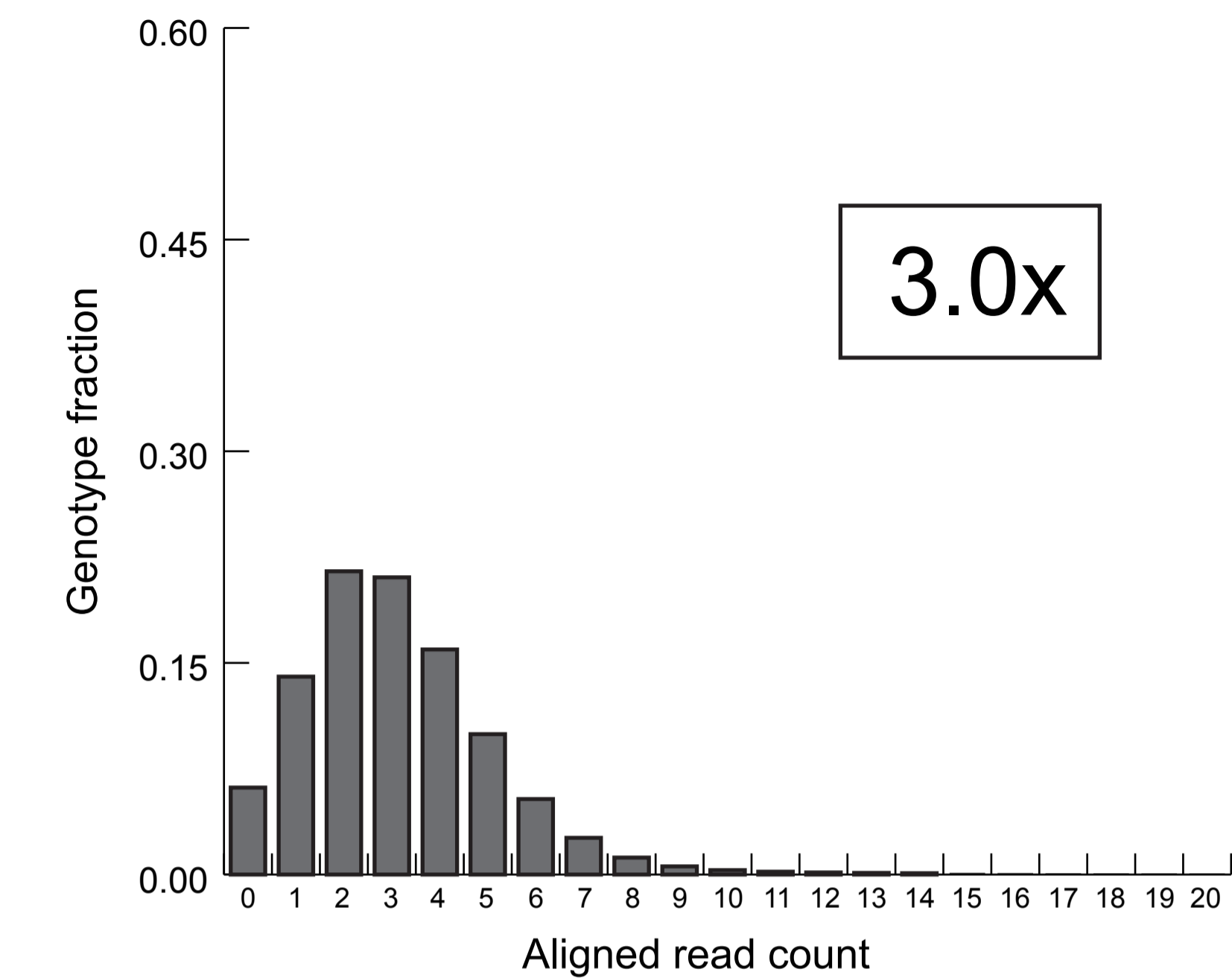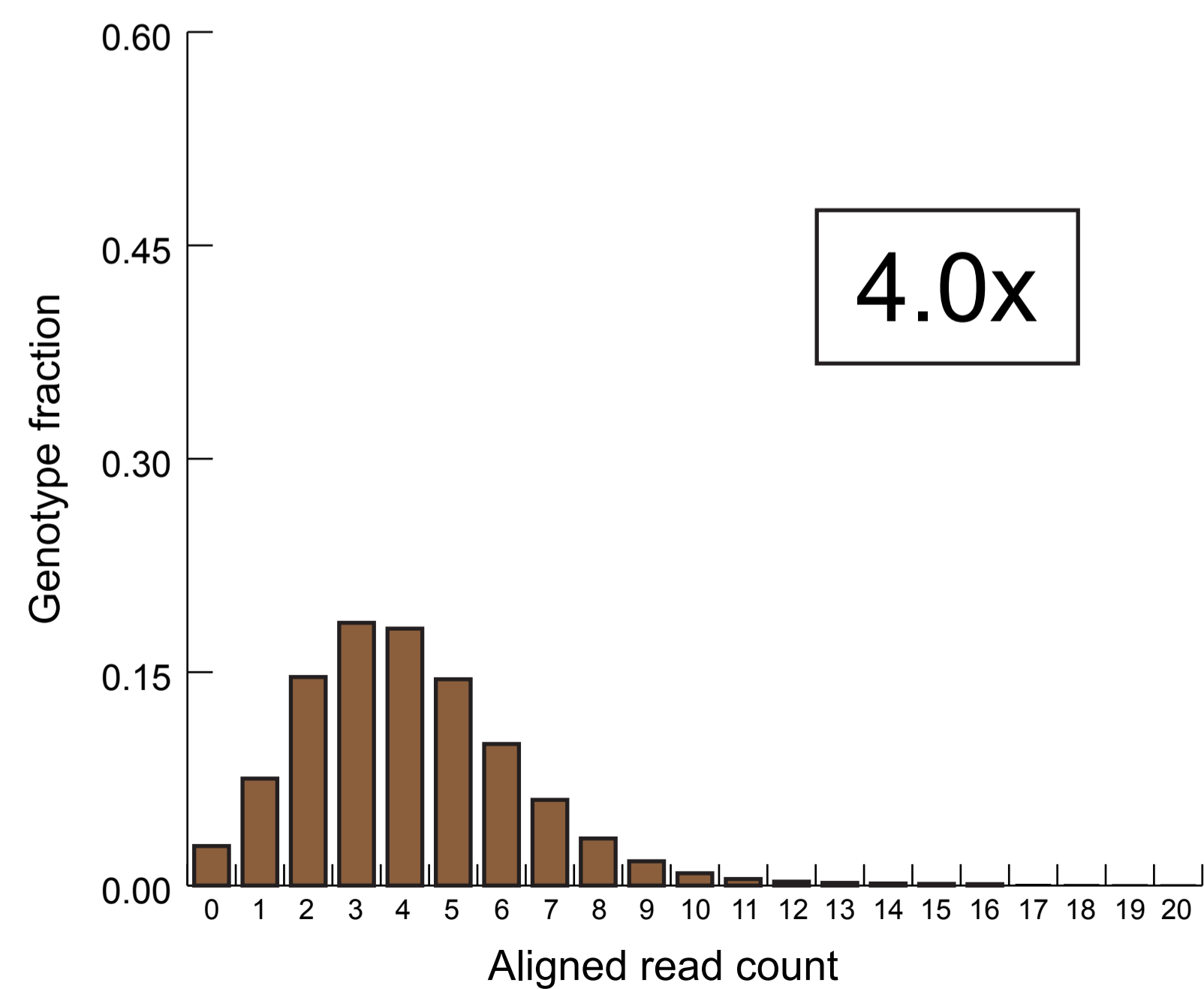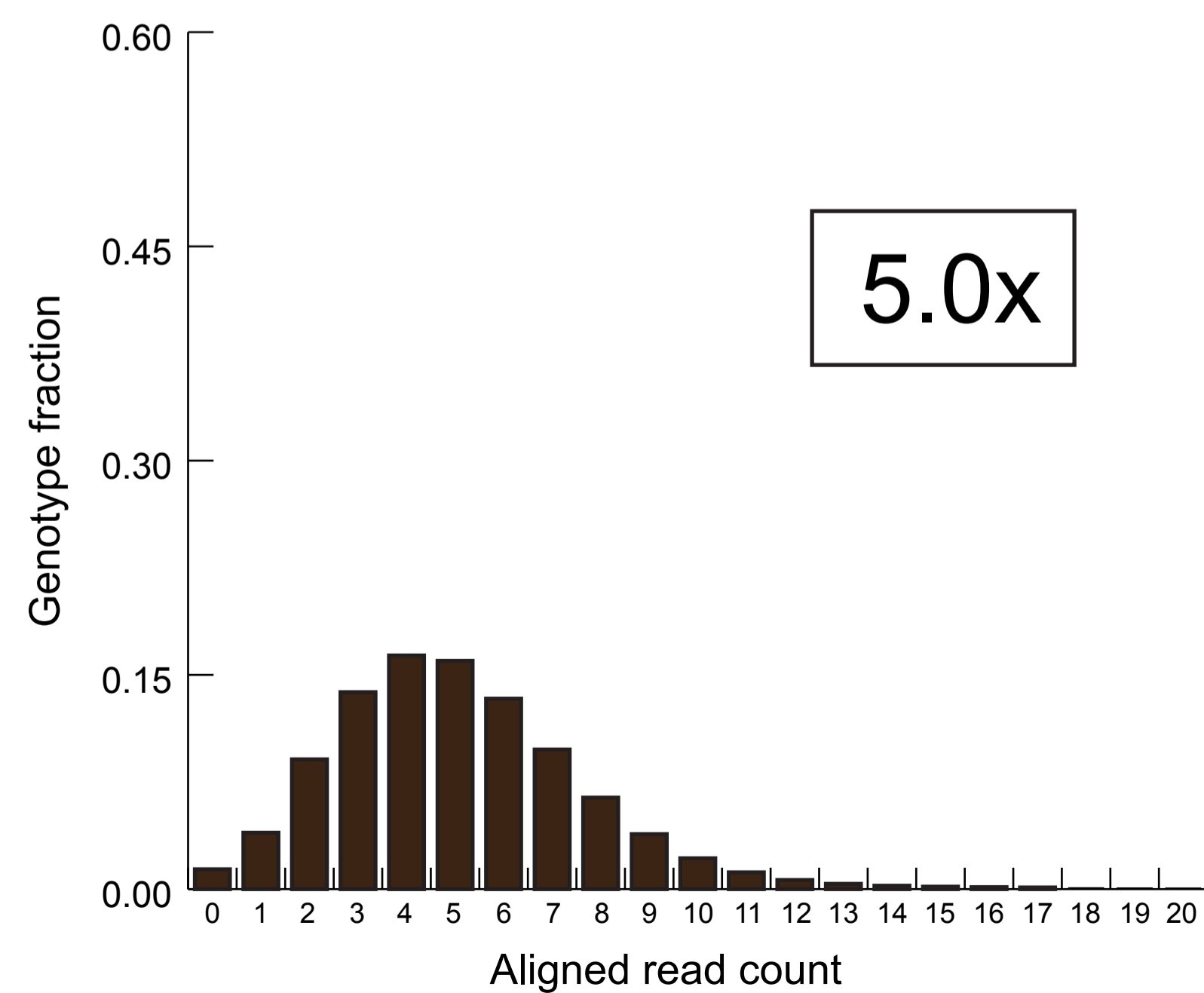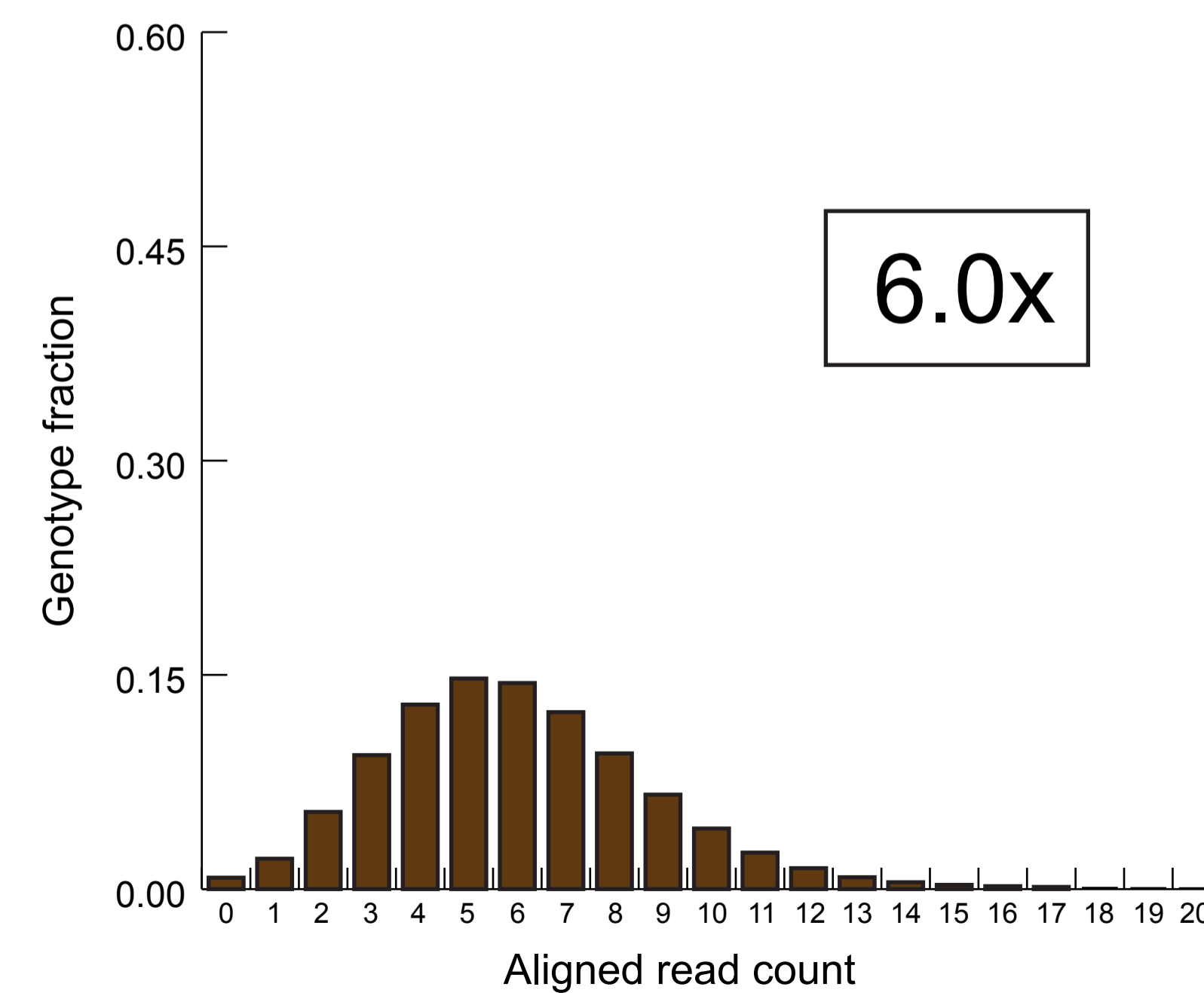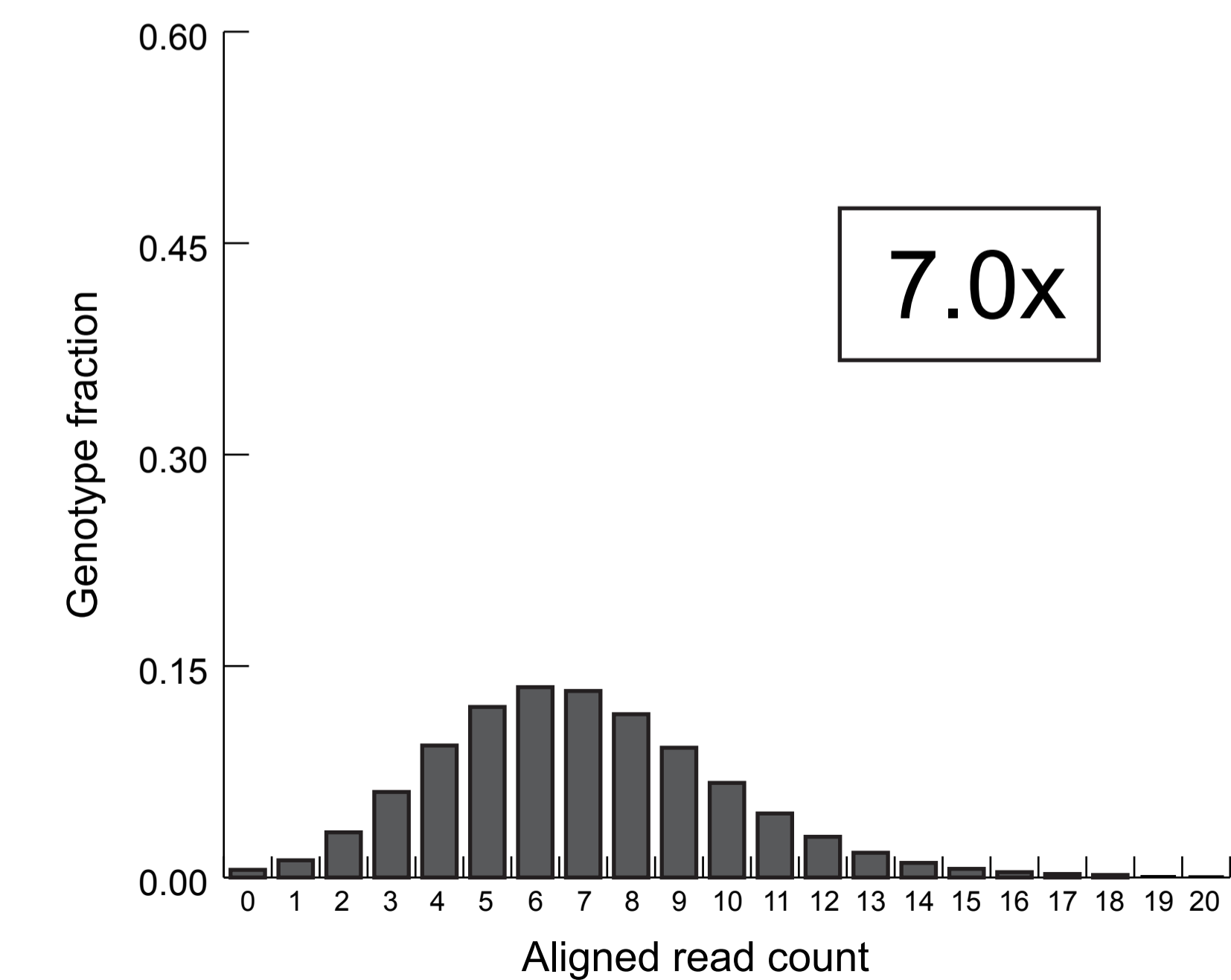

Supplement: Supplementary file 1 — Additional file 1: Figure S1. Distribution of aligned read per genotype of downsampled WGS of eight individuals. The x-axis represents the number of read counts aligned to the genotypes. The y-axis represents a fraction of genotypes from downsampled WGS of eight individuals. [file 40246_2021_357_MOESM1_ESM.pdf]

**a**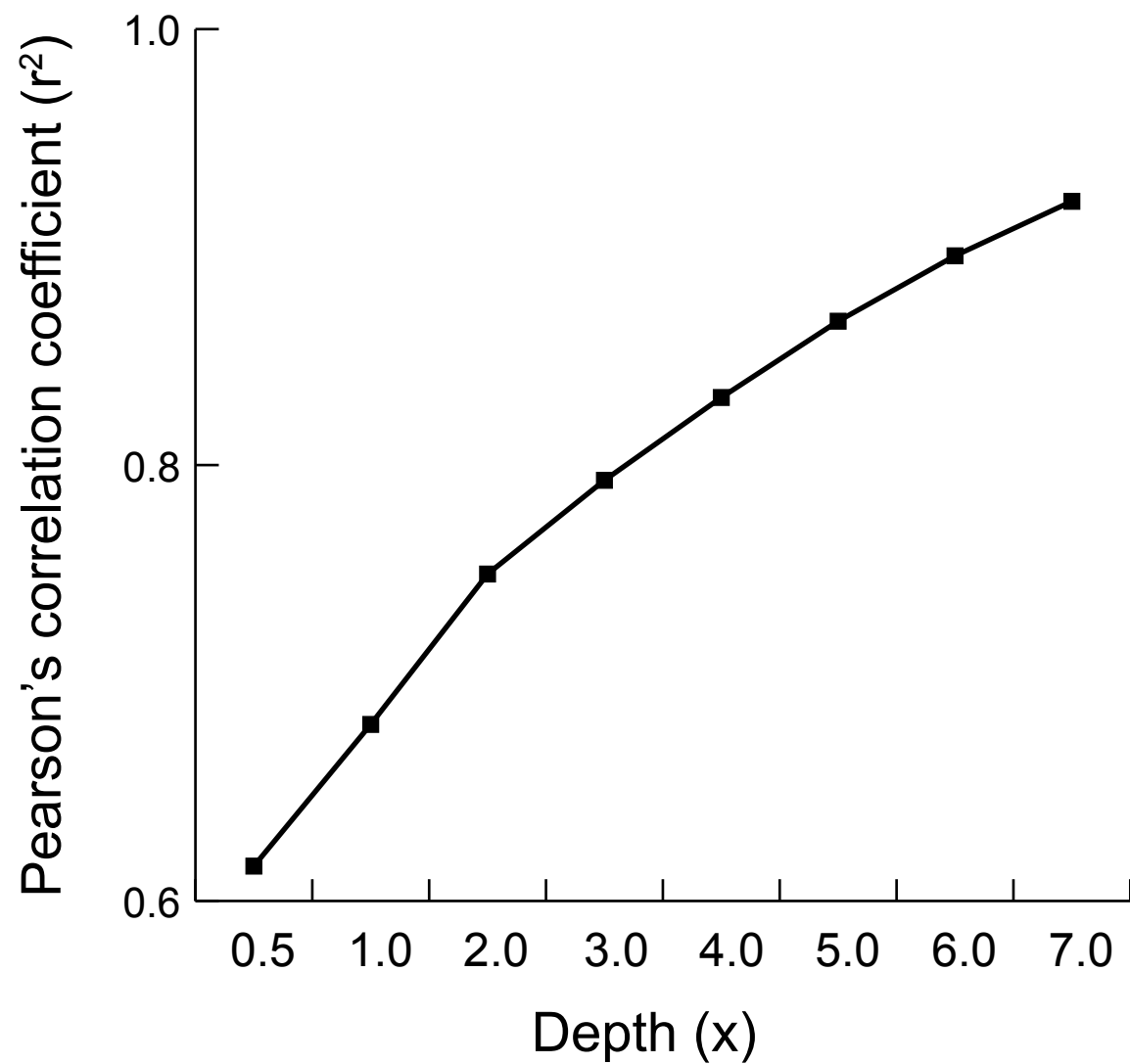**b**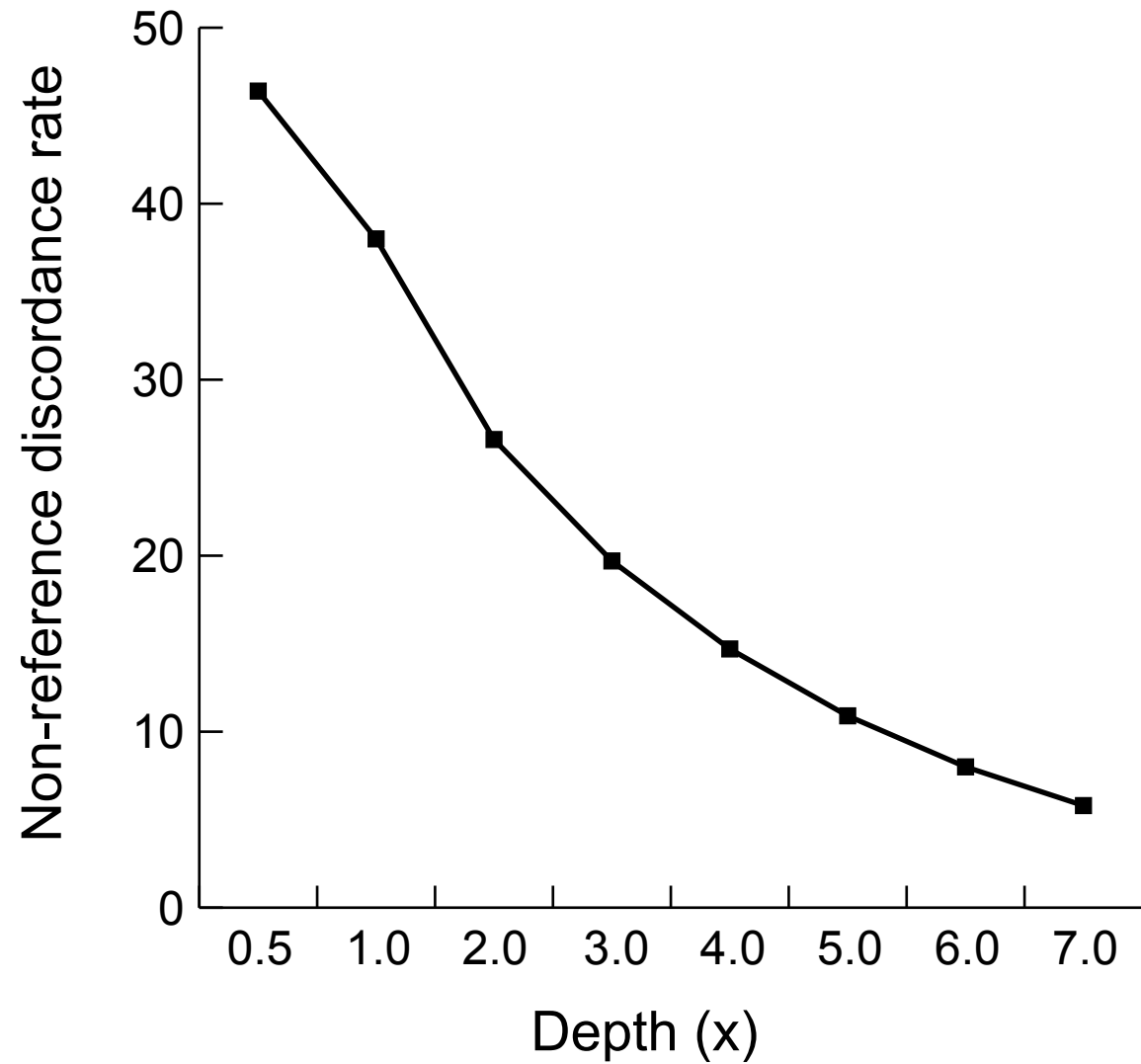

Supplement: Supplementary file 2 — Additional file 2: Figure S2. Genotype concordance between high-coverage genotypes and non-imputed genotypes. The x-axis represents each downsampled depths, and the y-axis represents a, Pearson’s correlation coefficient (R2), and b, Non-reference discordance rate. [file 40246_2021_357_MOESM2_ESM.pdf]

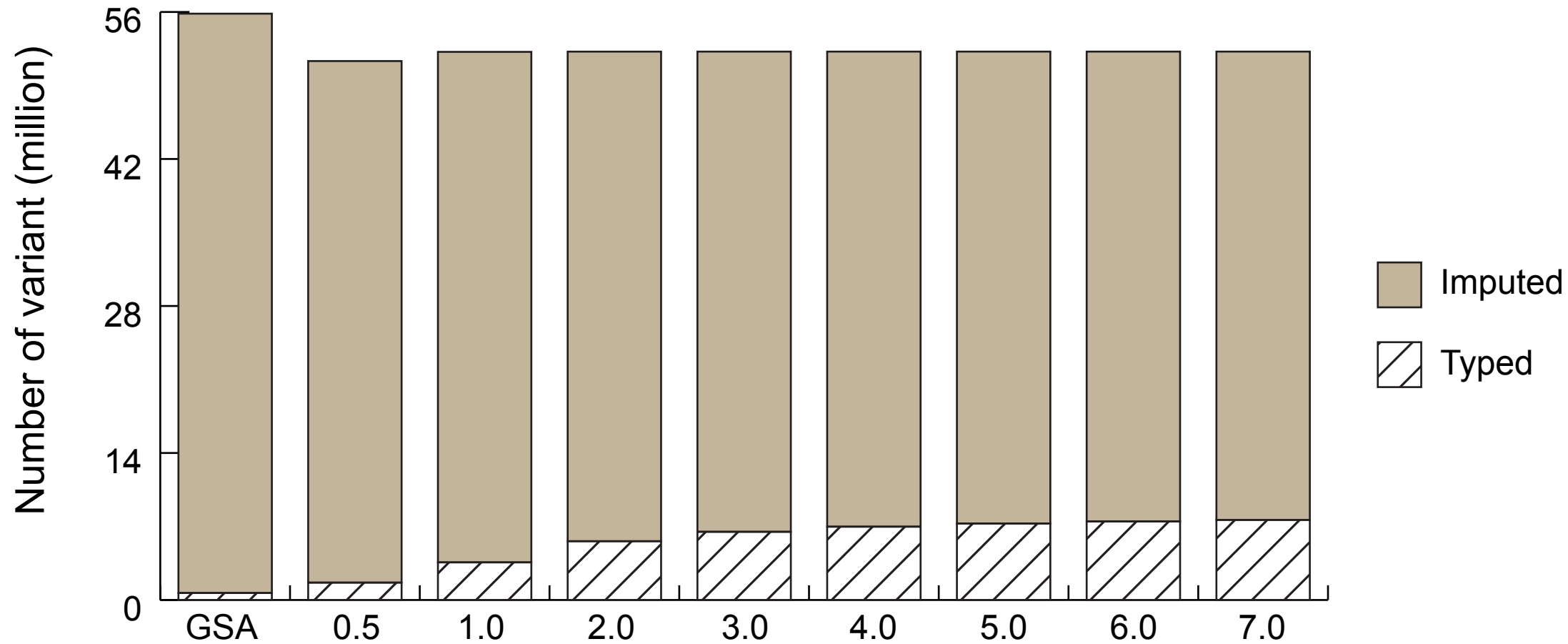

Supplement: Supplementary file 3 — Additional file 3: Figure S3. Number of typed and imputed variants in million from simulated array (GSA) and downsampled LPS from 0.5 × to 7.0 × . [file 40246_2021_357_MOESM3_ESM.pdf]

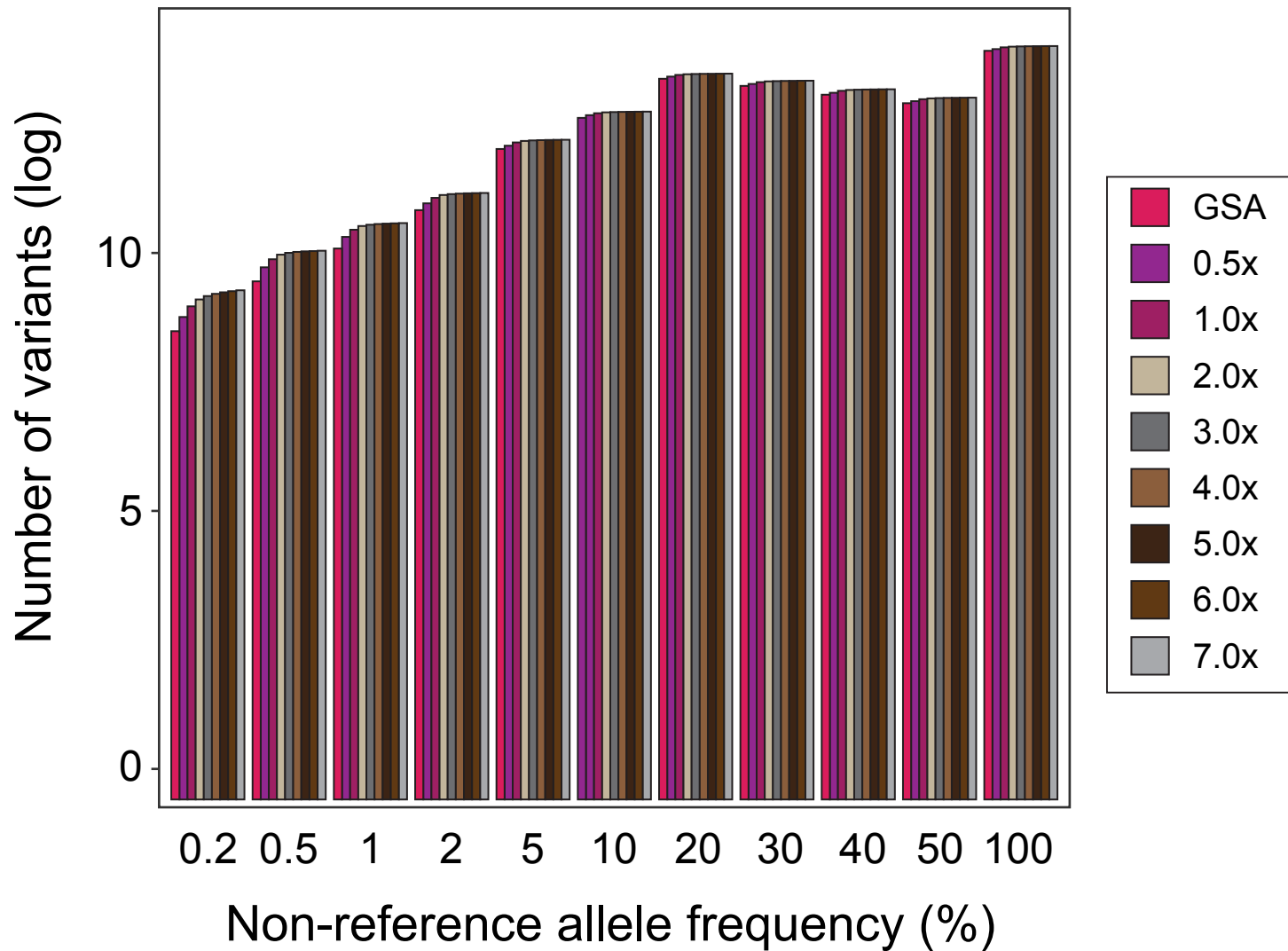

Supplement: Supplementary file 4 — Additional file 4: Figure S4. Number of variant with R2 > 0.8 from simulated array (GSA) and downsampled LPS from 0.5 × to 7.0 × across each frequency bins. The x-axis represents non-reference allele frequency (AF) of East Asian population derived from the gnomAD v3.1. The y-axis is the number of variants in log scale. [file 40246_2021_357_MOESM4_ESM.pdf]

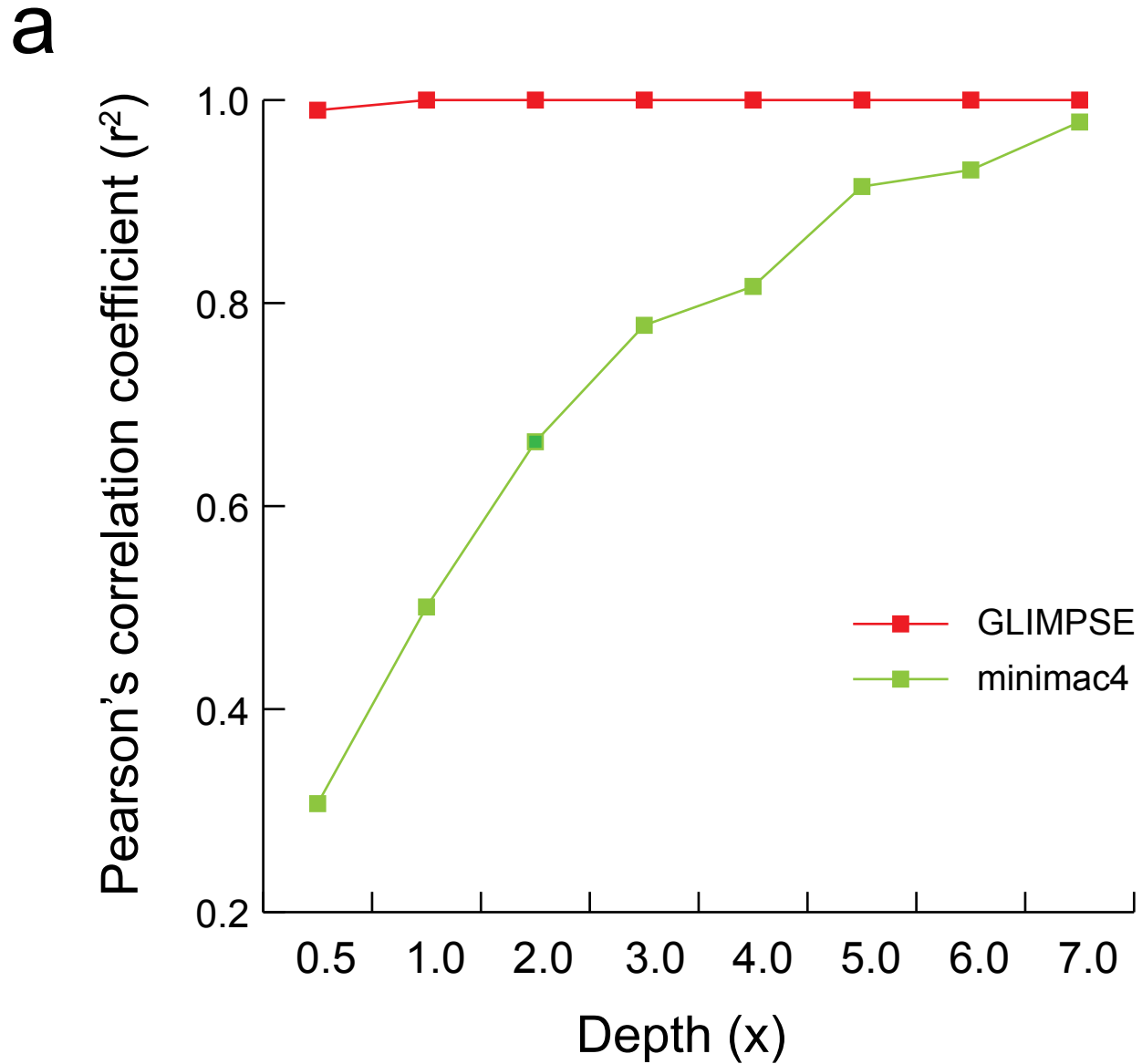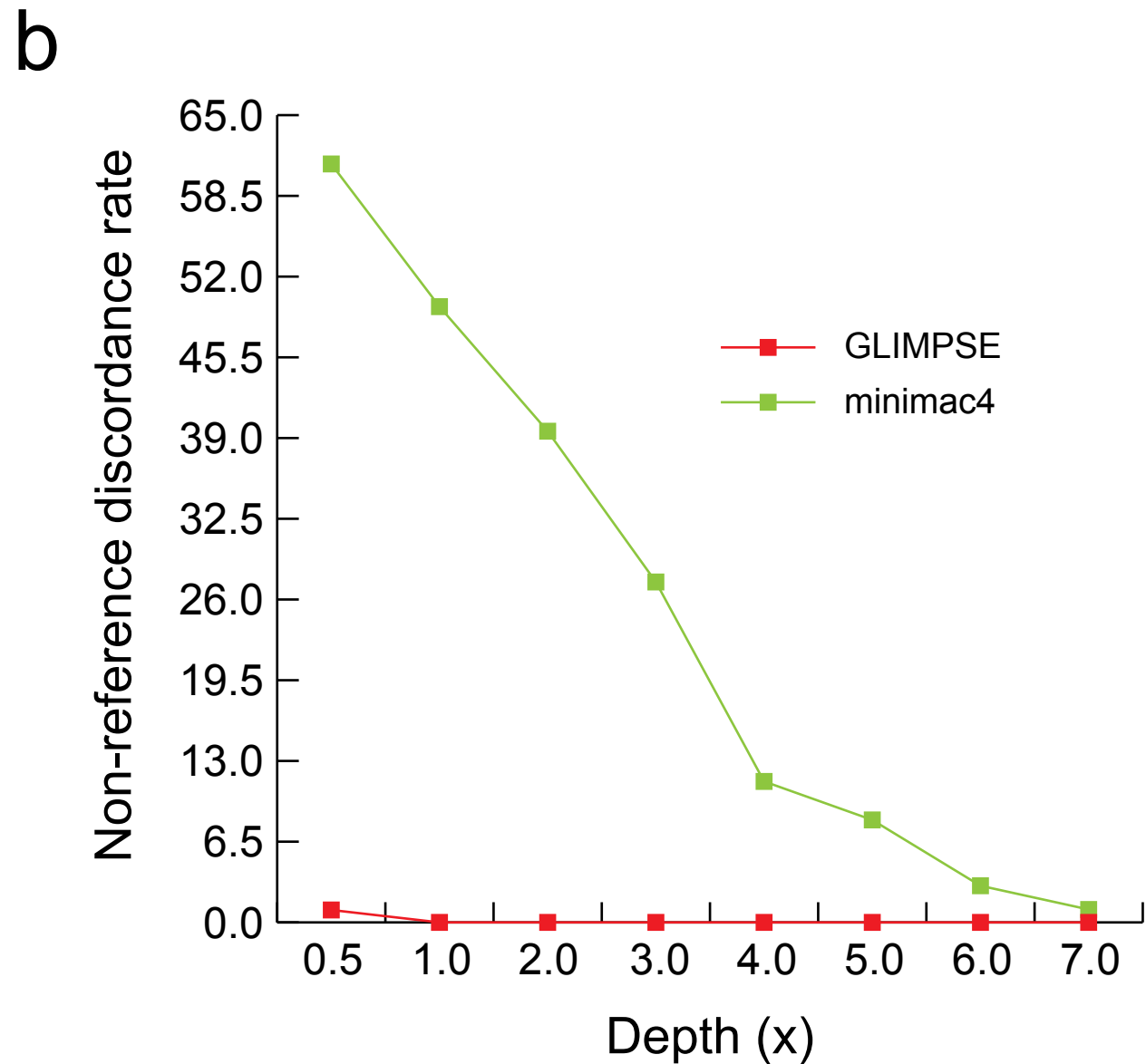

Supplement: Supplementary file 5 — Additional file 5: Figure S5. Comparison of imputation approach using 20 PD-associated SNPs. Red indicates imputation using GLIMPSE, and green indicates haplotype phasing using Eagle v2.4 and Minimac4 for imputation. a, Pearson’s correlation coefficient (R2) and b, Non-reference discordance rate. [file 40246_2021_357_MOESM5_ESM.pdf]

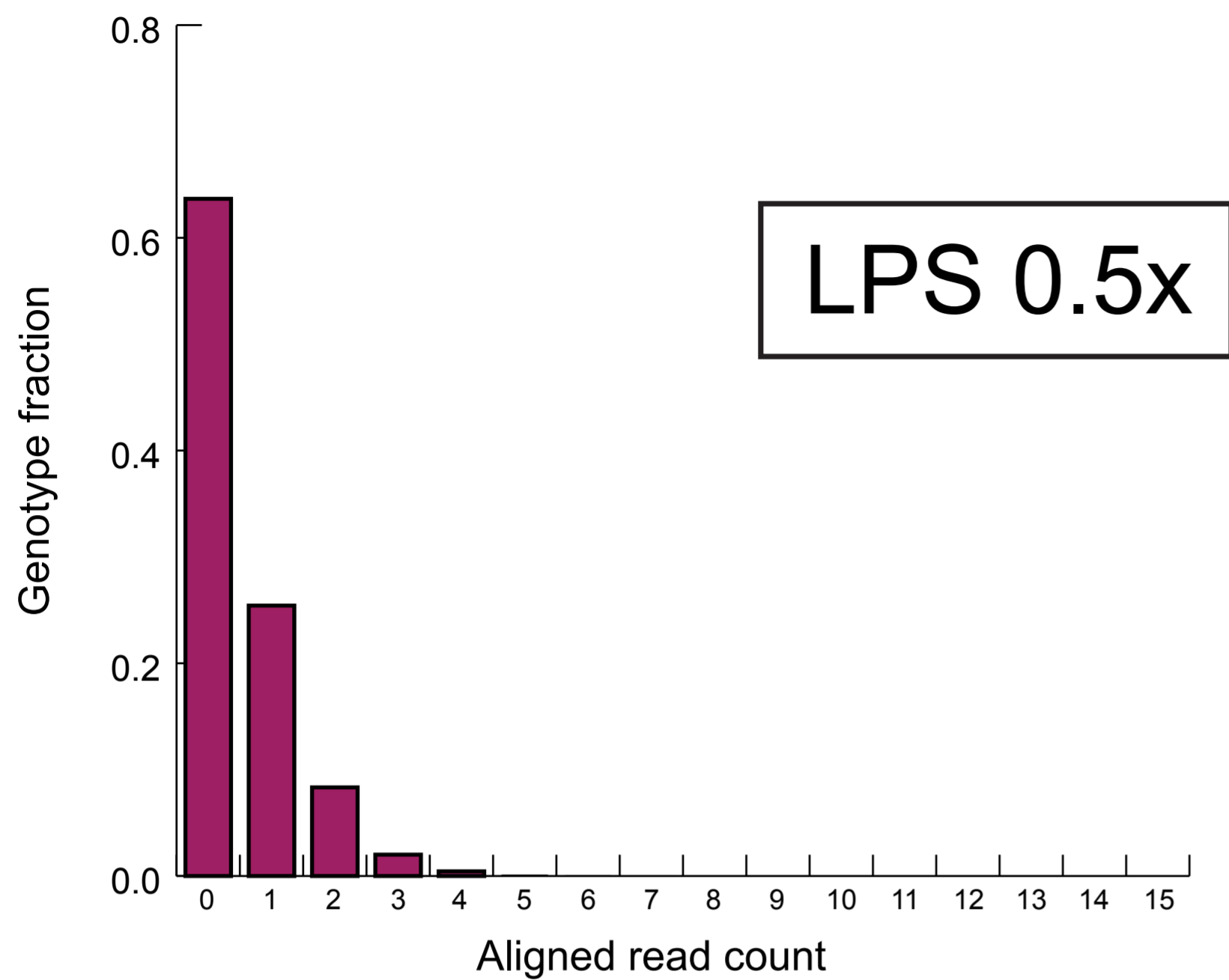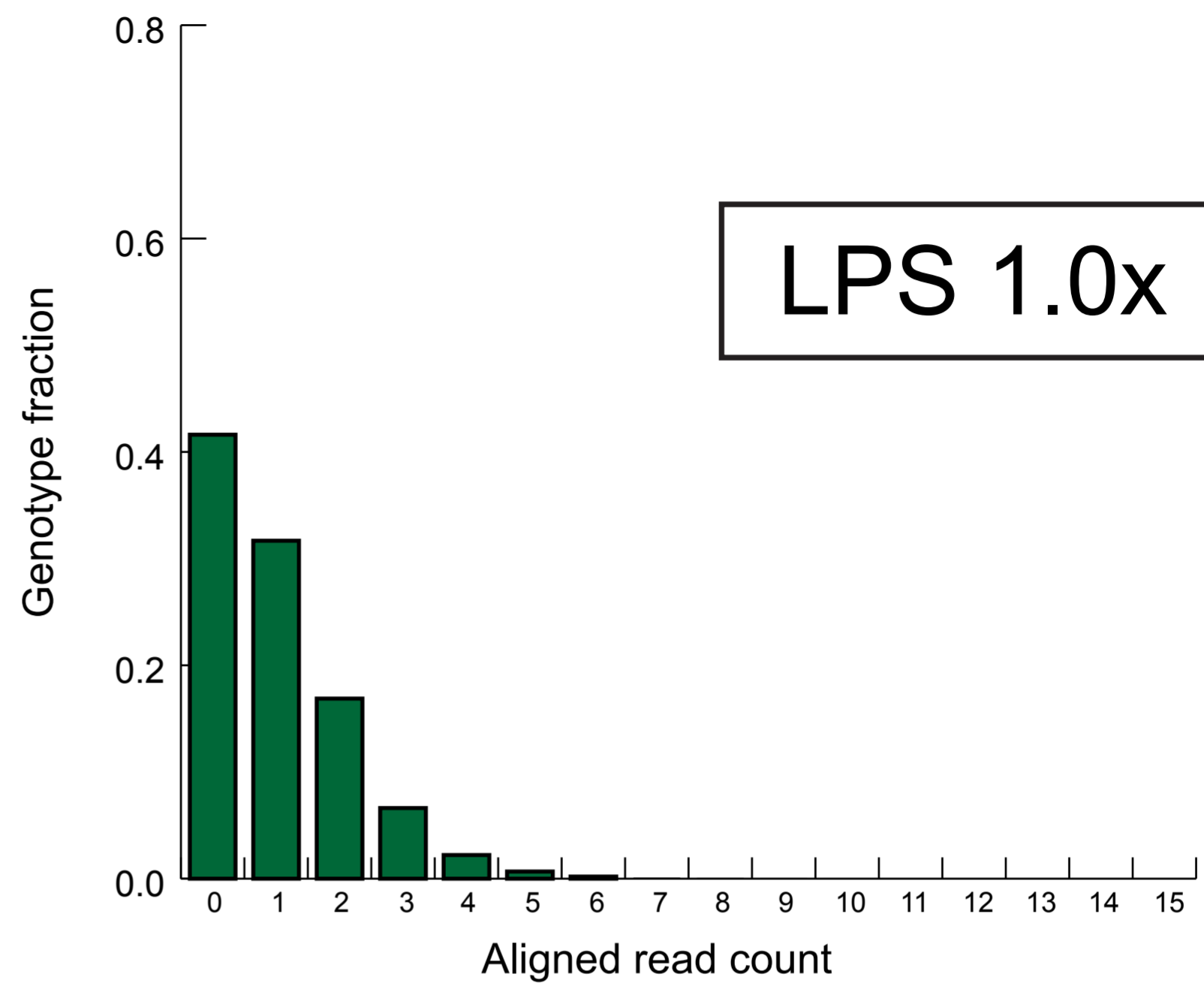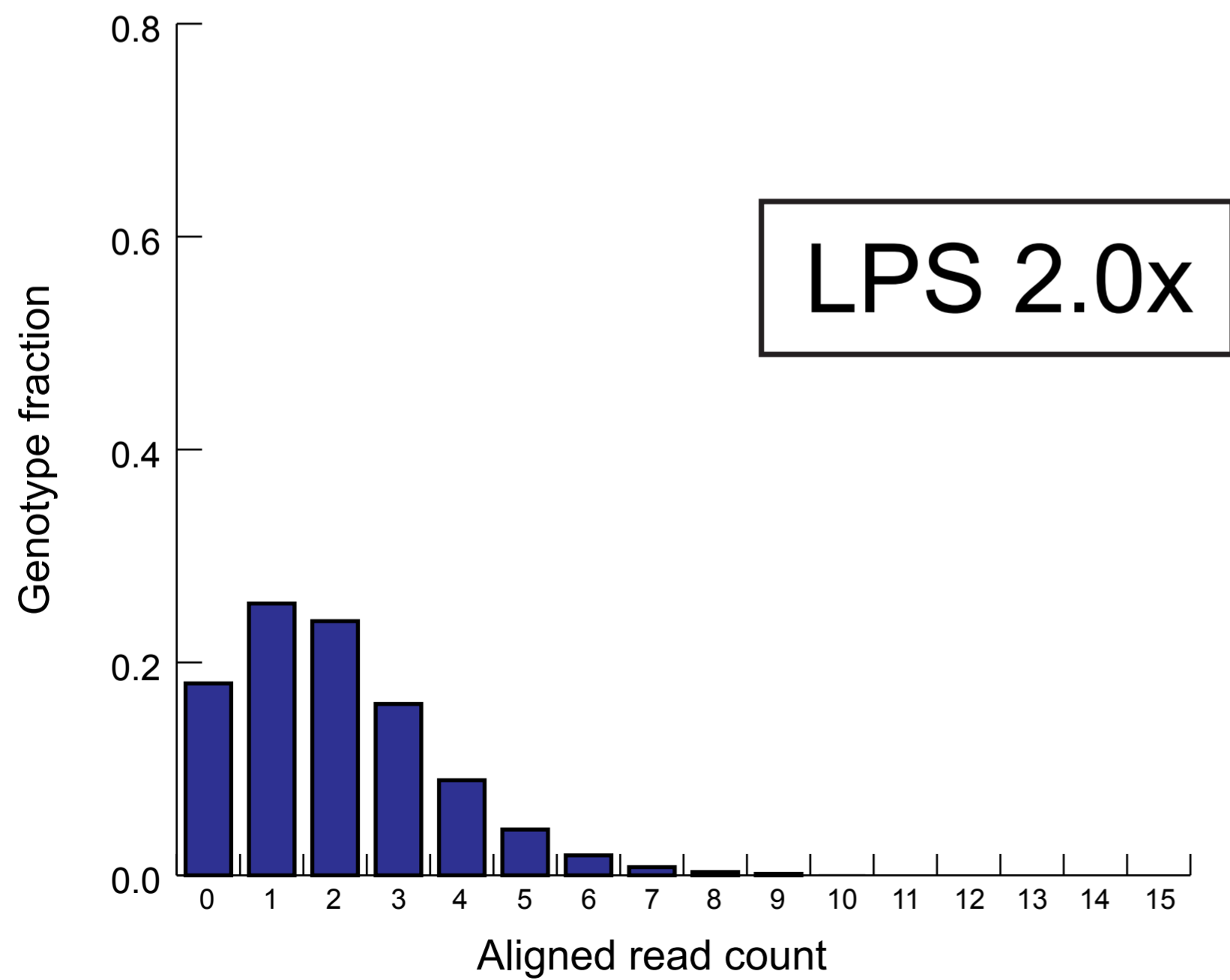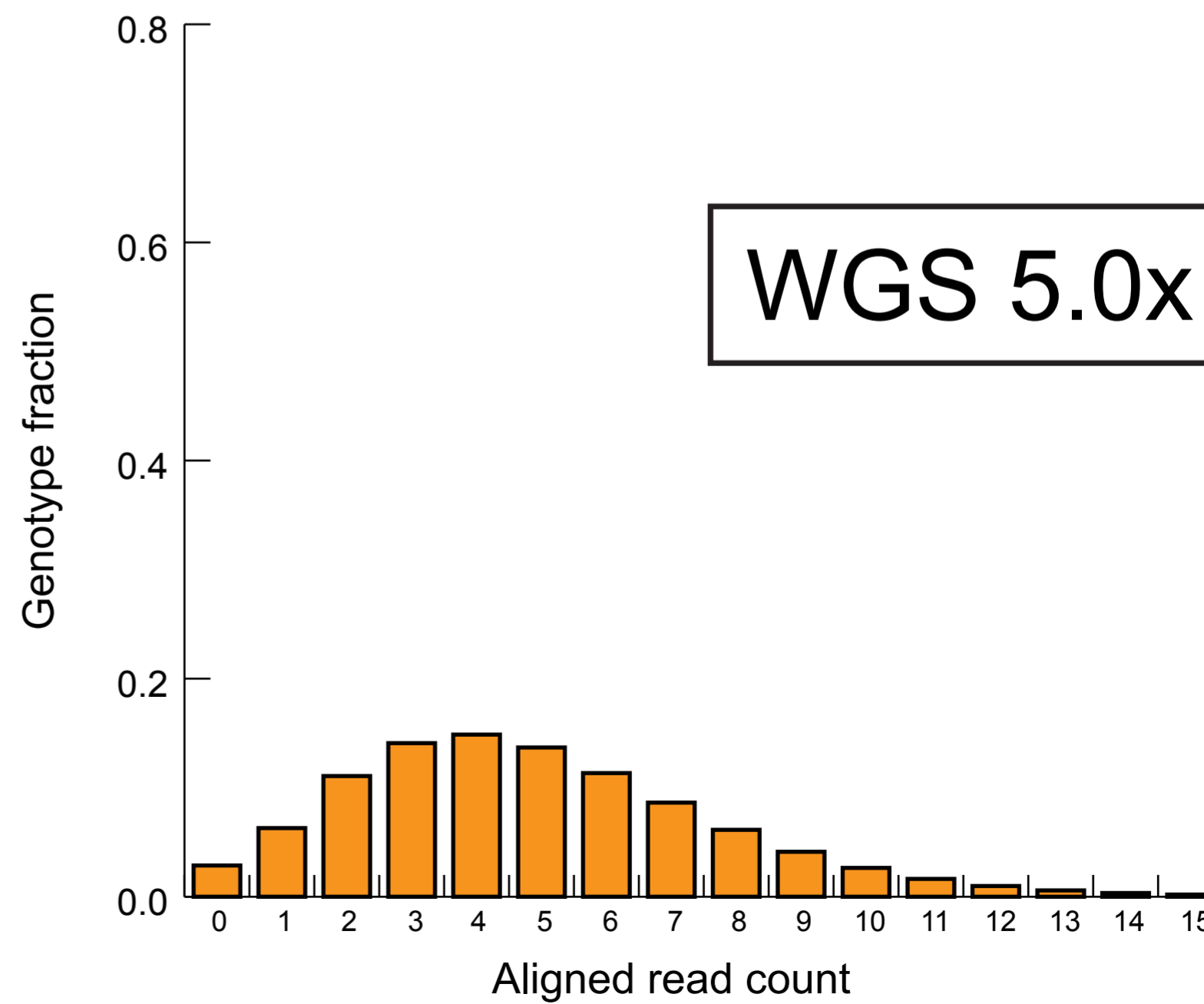

Supplement: Supplementary file 6 — Additional file 6: Figure S6. Distribution of aligned read per genotype of downsampled WGS of 188 individuals. The x-axis represents the number of read counts aligned to the genotypes. The y-axis represents a fraction of genotypes from downsampled WGS of 188 individuals. [file 40246_2021_357_MOESM6_ESM.pdf]

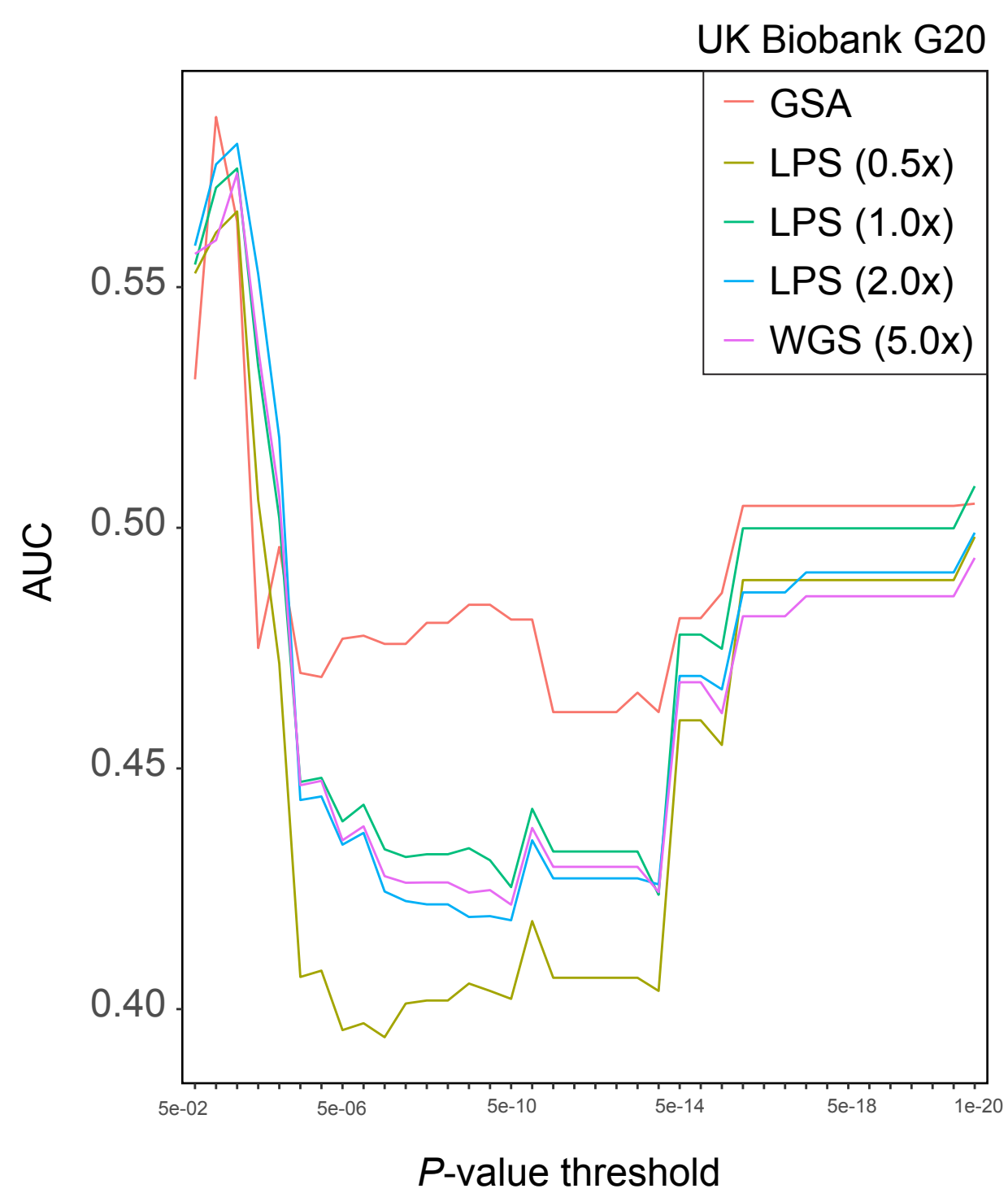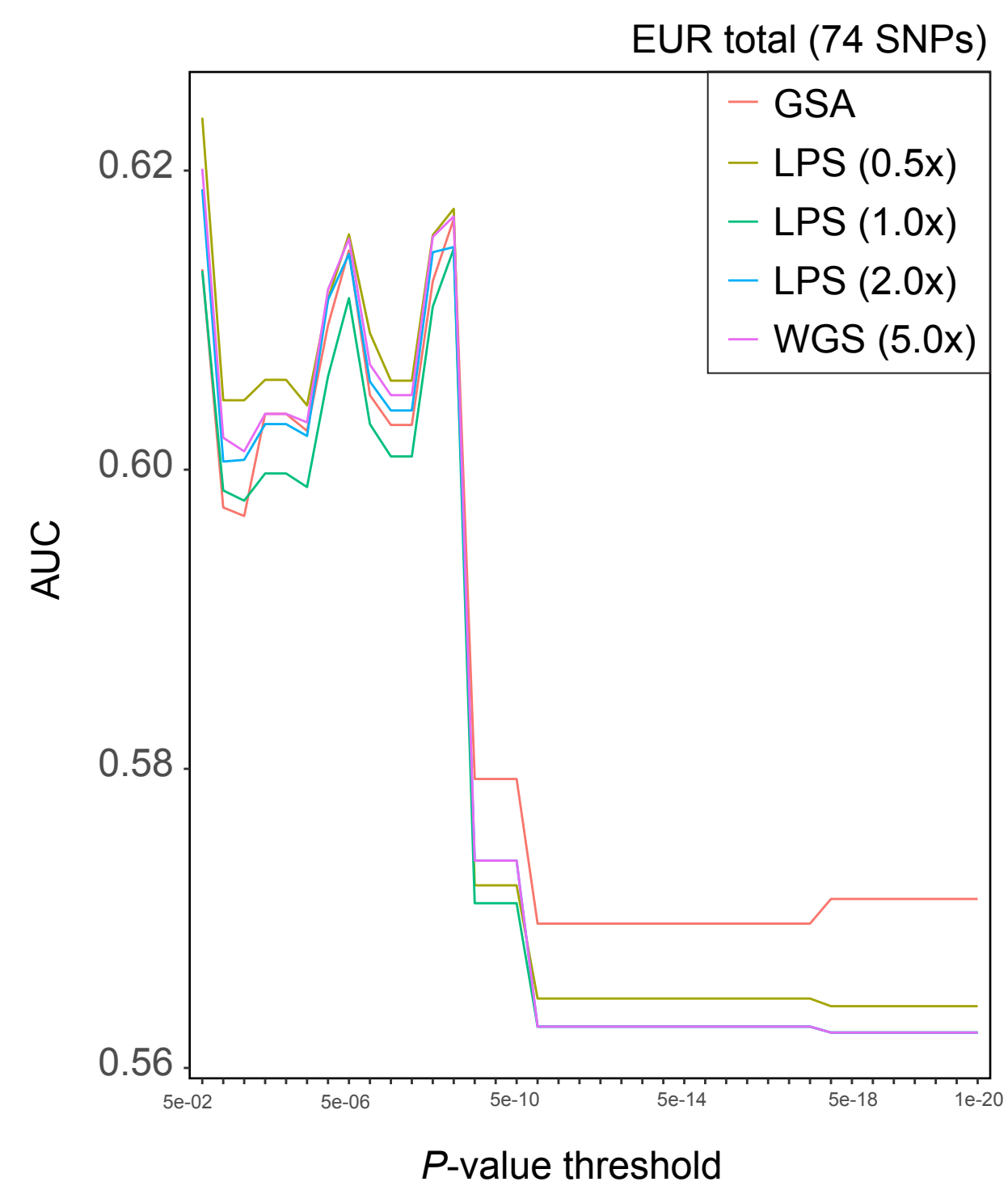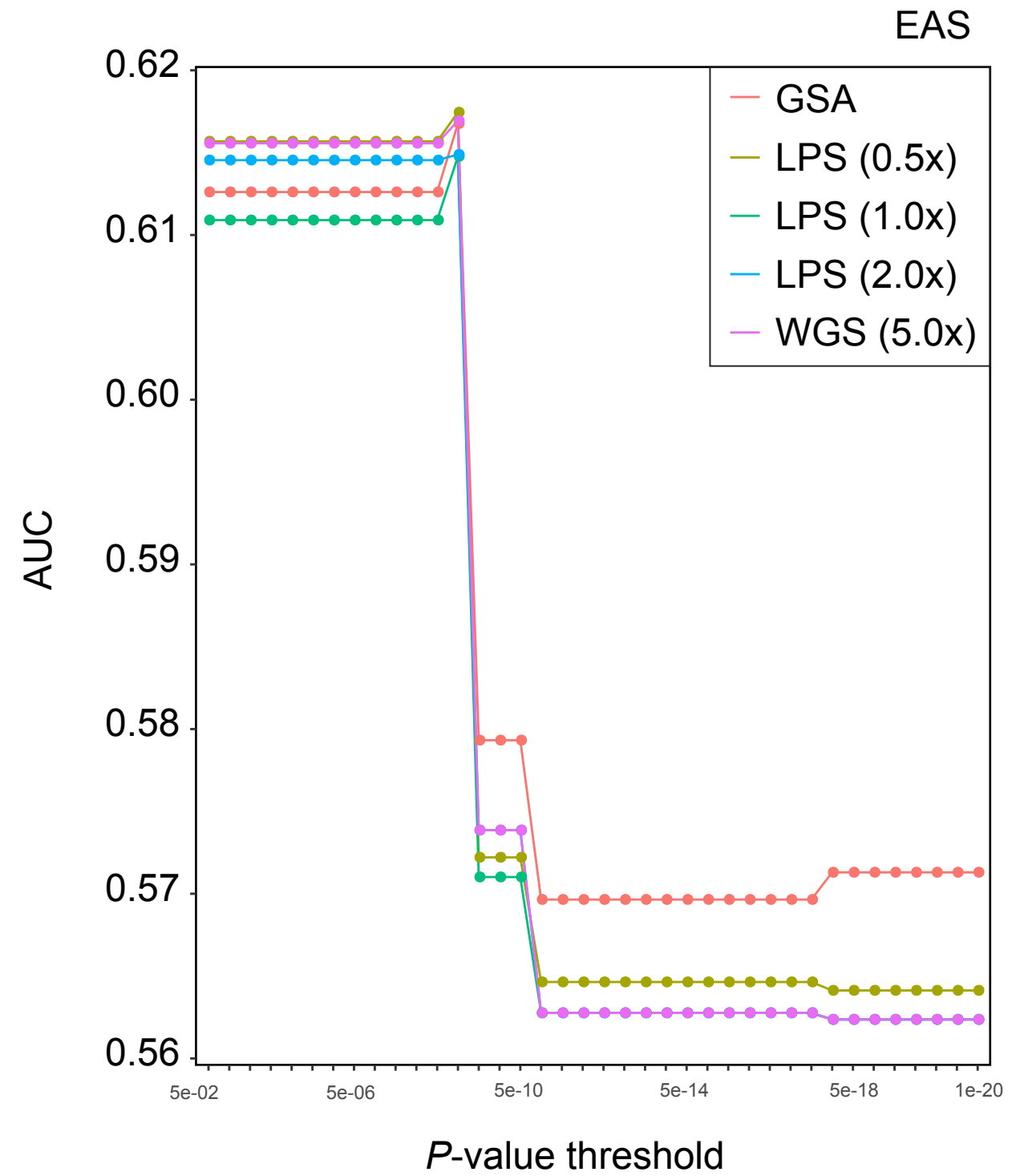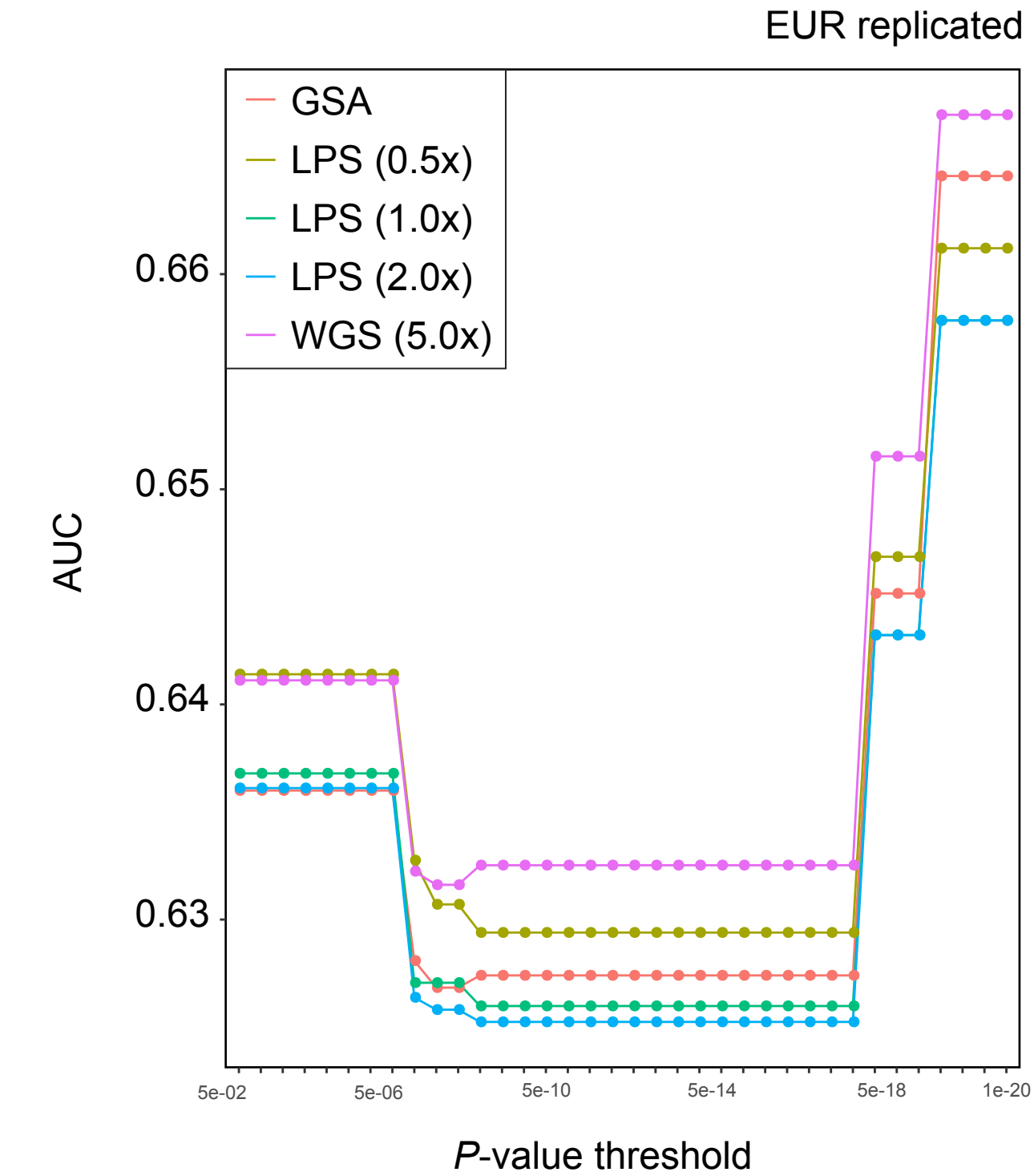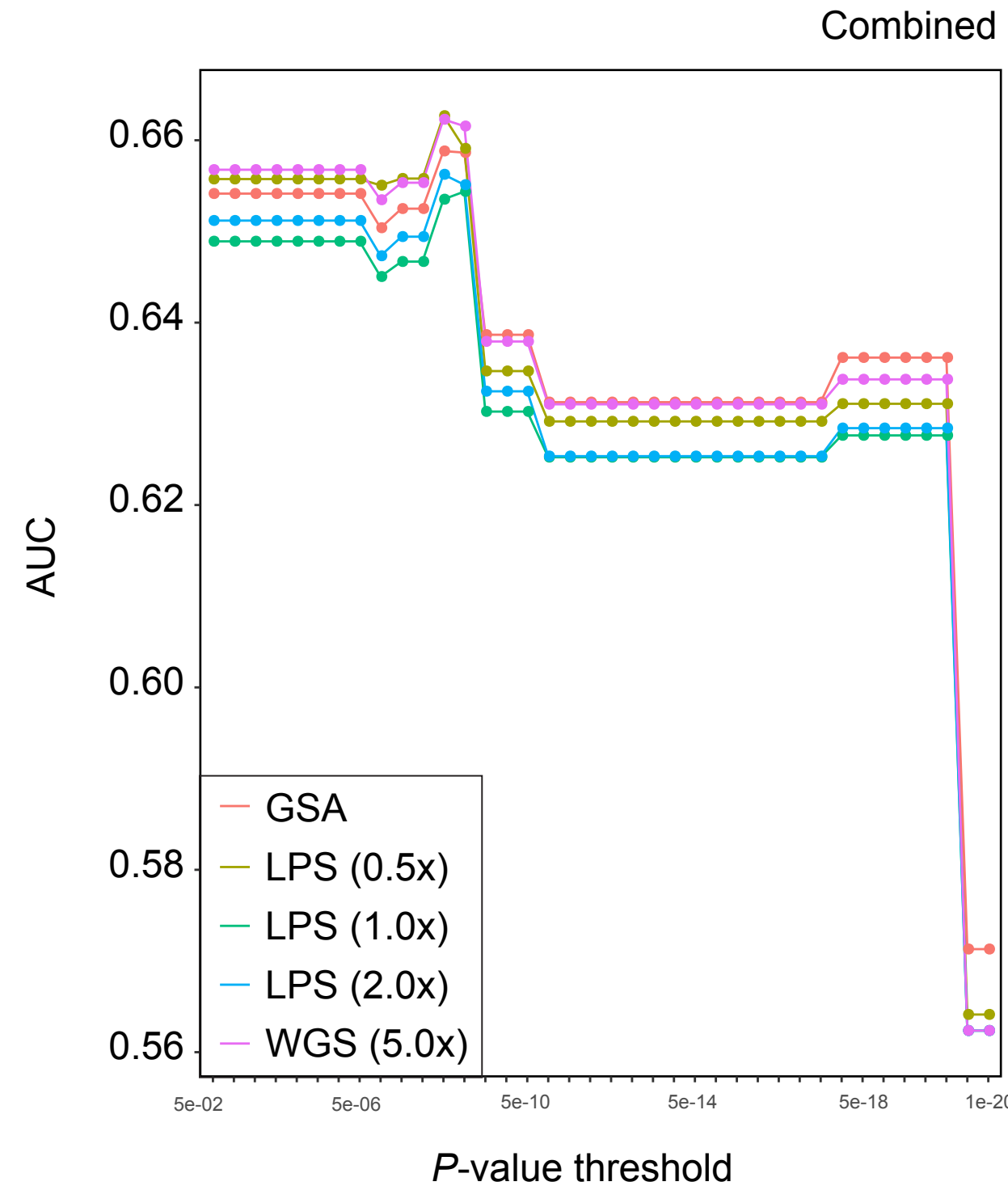

Supplement: Supplementary file 7 — Additional file 7: Figure S7. AUC of 5 different PRS sets at each P value threshold. After LD clump, multiple P value thresholds were set to assess AUC values. The x-axis represents significance thresholds from 5.00 × 10^–2^ to 1.00 × 10^–20^, and the y-axis represent AUC. [file 40246_2021_357_MOESM7_ESM.pdf]

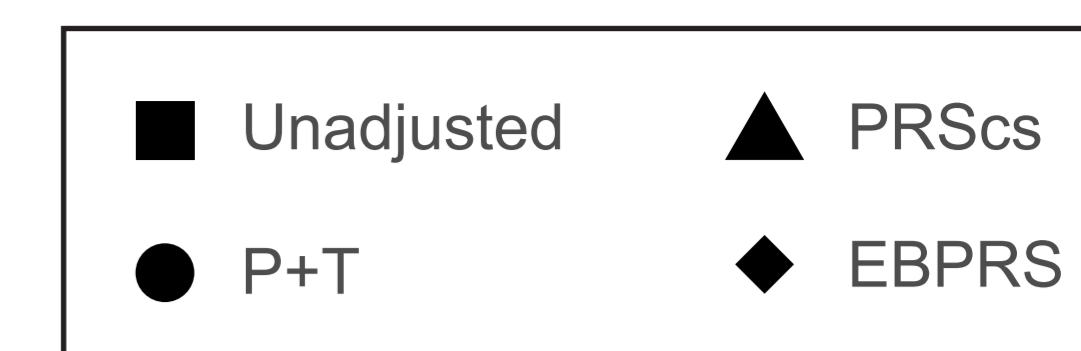

LPS 0.5x

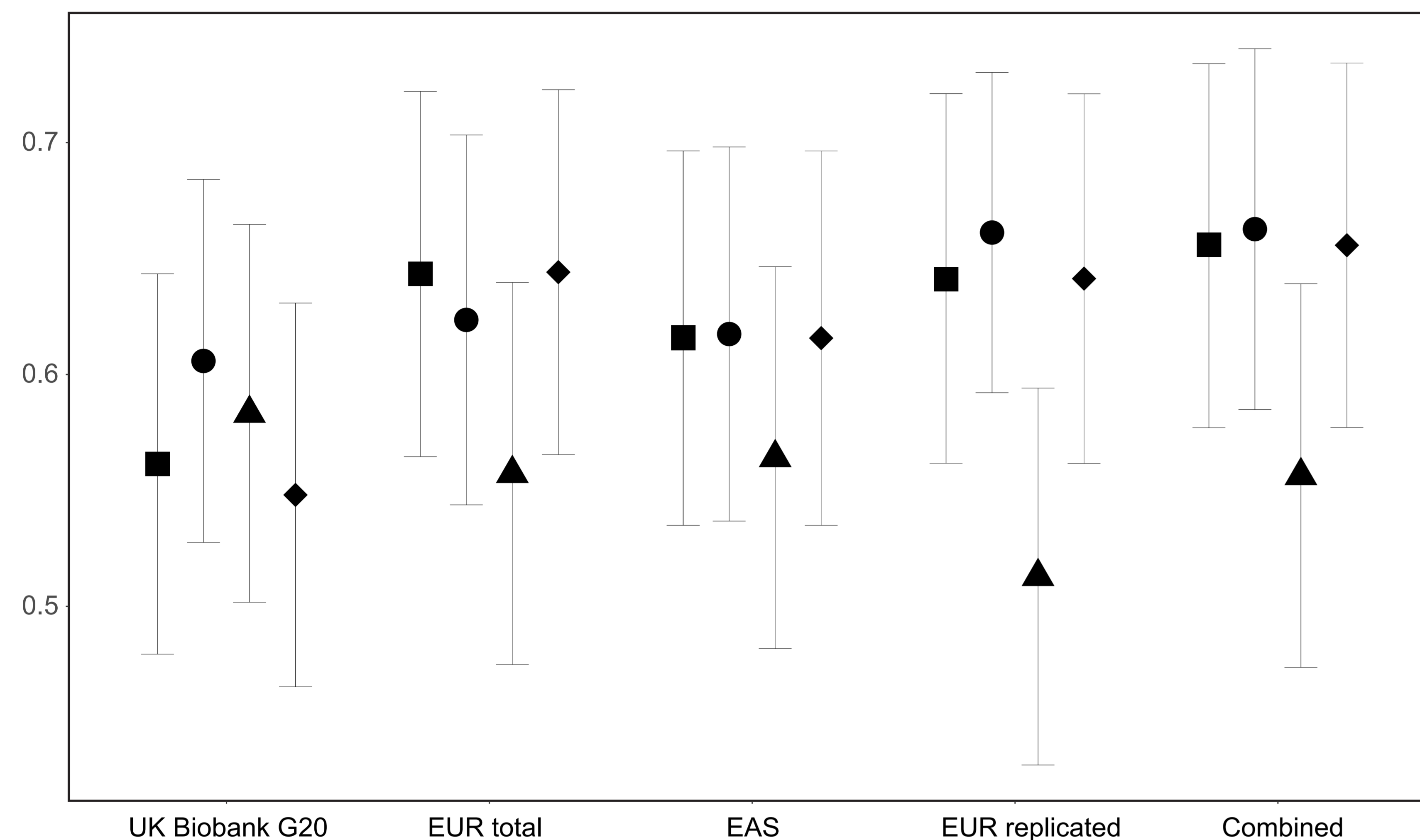

LPS 1.0x

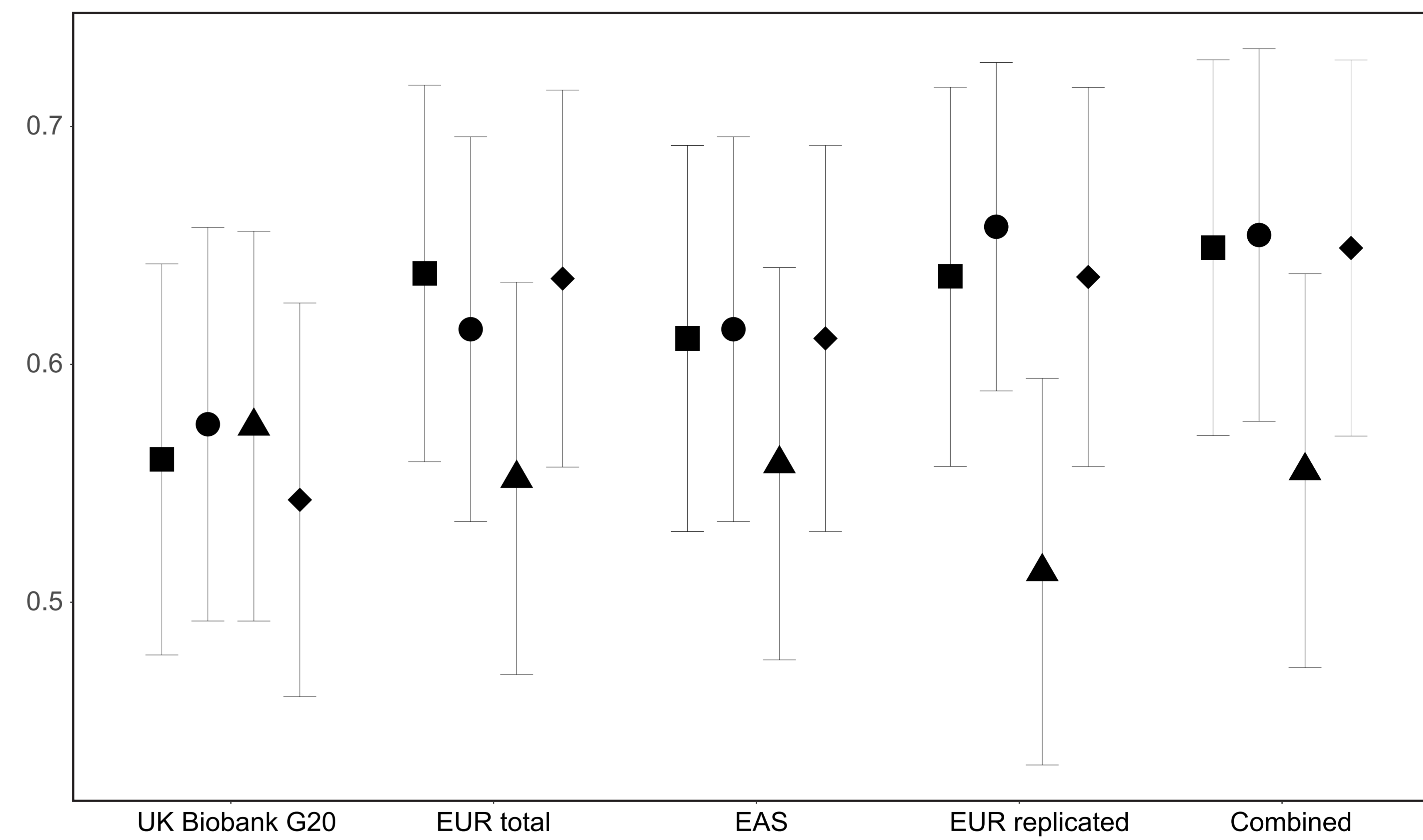

LPS 2.0x

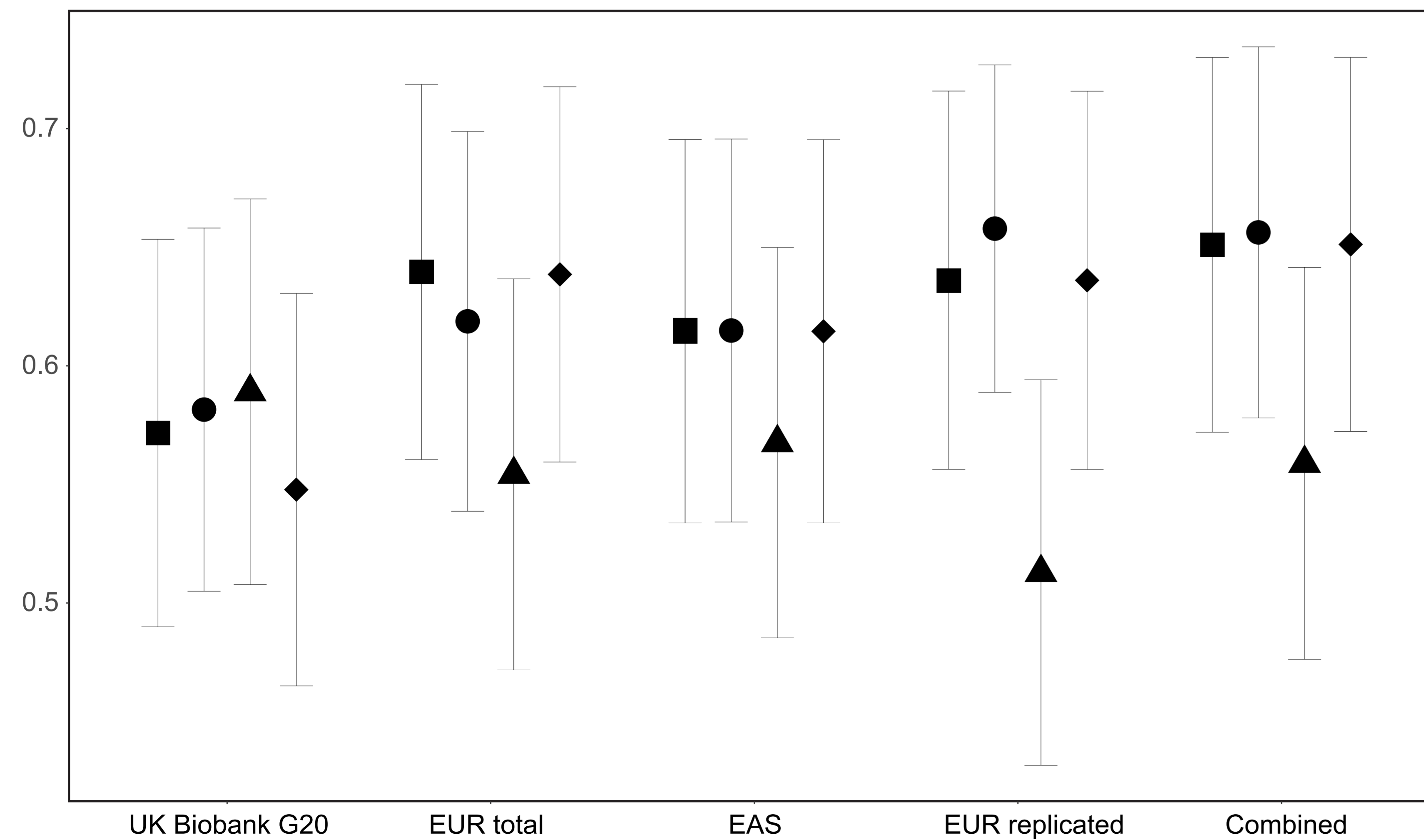

WGS (5.0x)

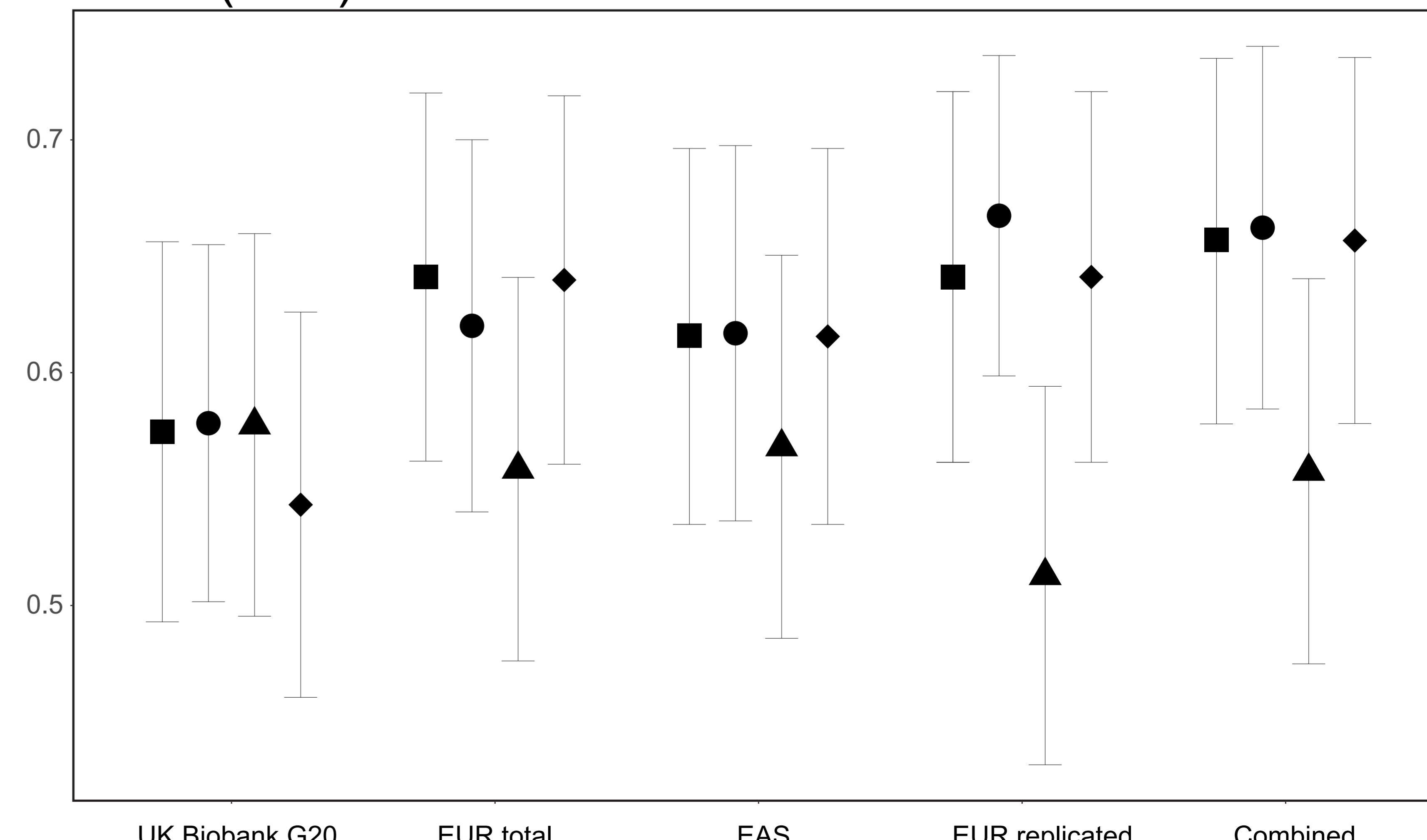

AUC

Supplement: Supplementary file 8 — Additional file 8: Figure S8. Assessment of PRS models based on SNP sets from GWAS in European and East Asian populations using LPS. Evaluation of 5 different PRS models using 4 different PRS approaches based on data of GSA followed by imputation. The x-axis presents PRS models: UK Biobank G20; GWAS summary statistics of PD from the UK Biobank study of European populations, EUR total; 74 previously identified PD-associated SNPs in European populations, EAS; 11 genome-wide significant SNPs in a meta-GWAS of East Asians (P < 5.00 × 10^–8^), EUR replicated; 9 SNPs in EUR total that were replicated in a meta-GWAS (P < 1.00 × 10^–5^), and Combined; 16 SNPs of EAS and EUR replicated that were LD clumped. A total of 4 different approaches for PRS calculations were used: unadjusted, P + T, PRScs, and EB-PRS. The area under curve (AUC) with 95% confidence intervals is shown in the y-axis. [file 40246_2021_357_MOESM8_ESM.pdf]

0.5x

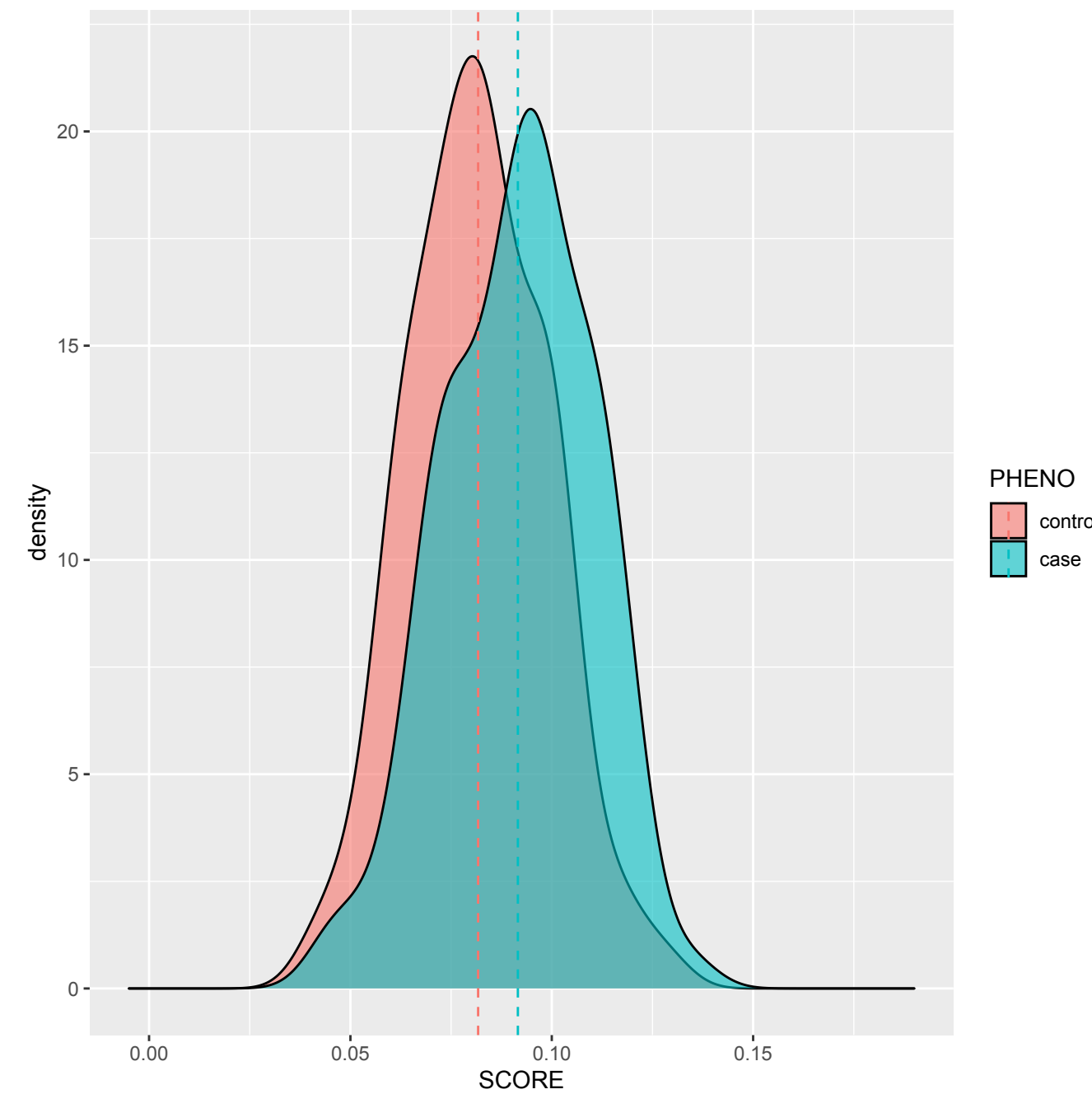

1.0x

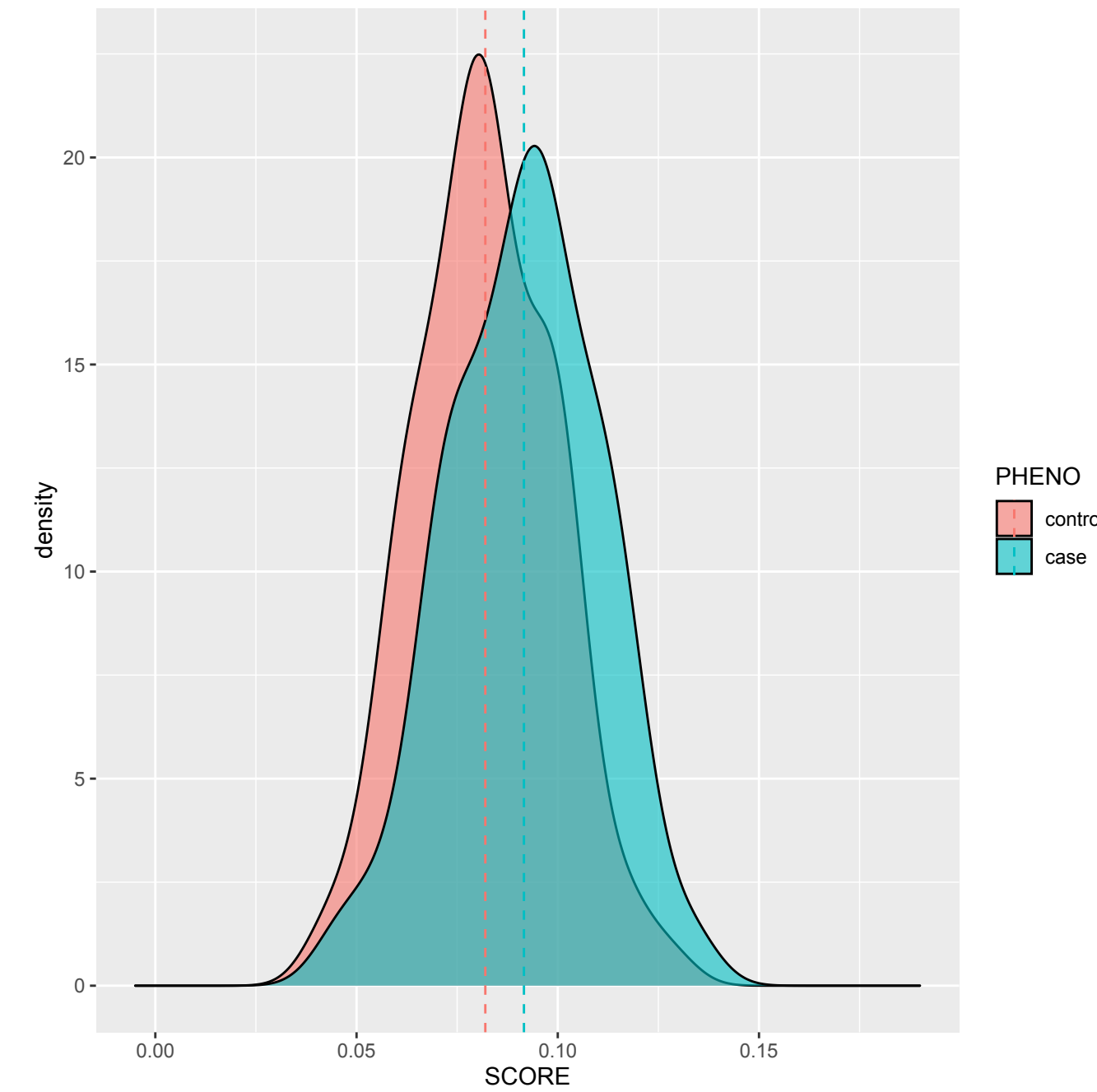

2.0x

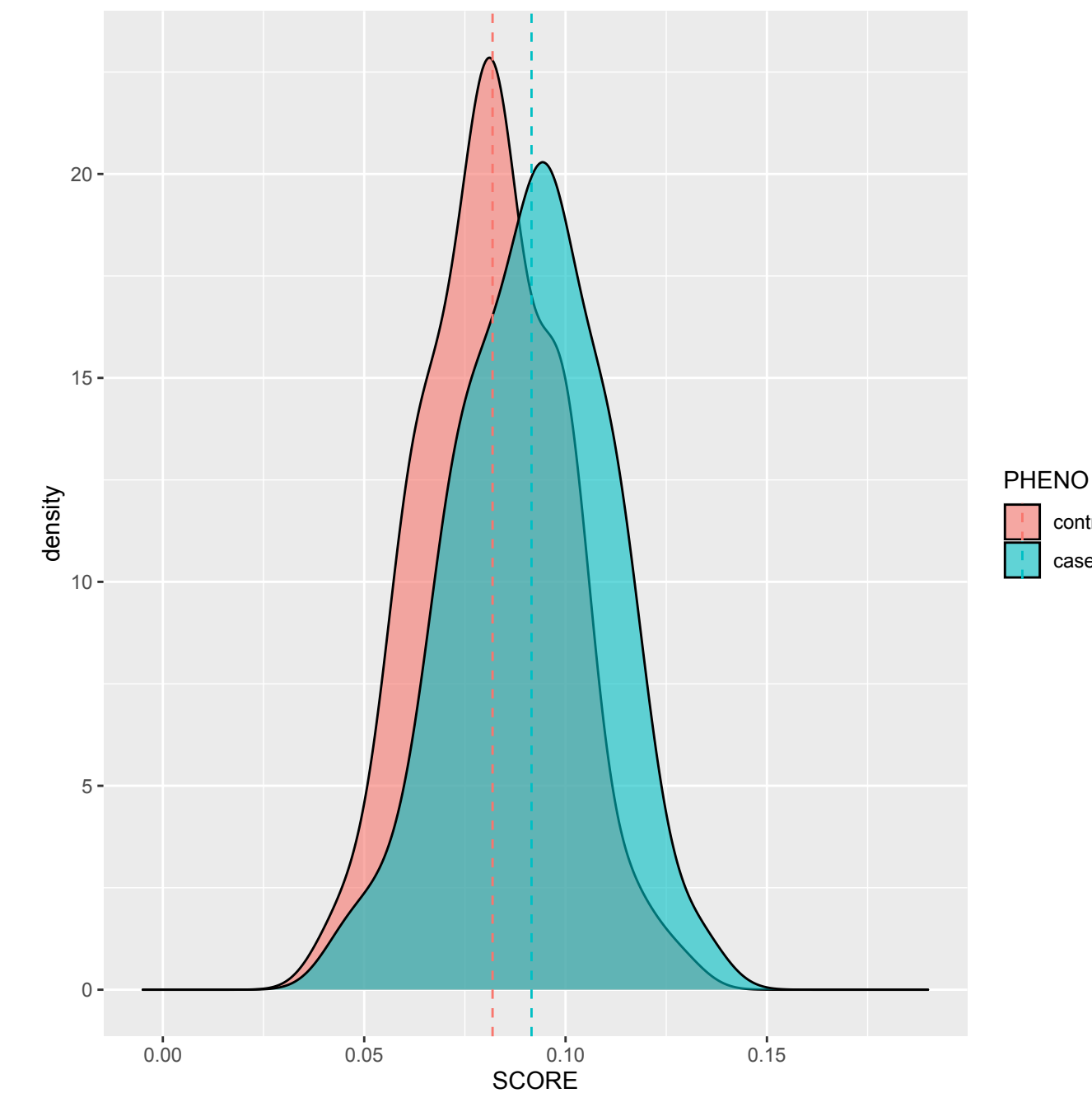

WGS 5.0x

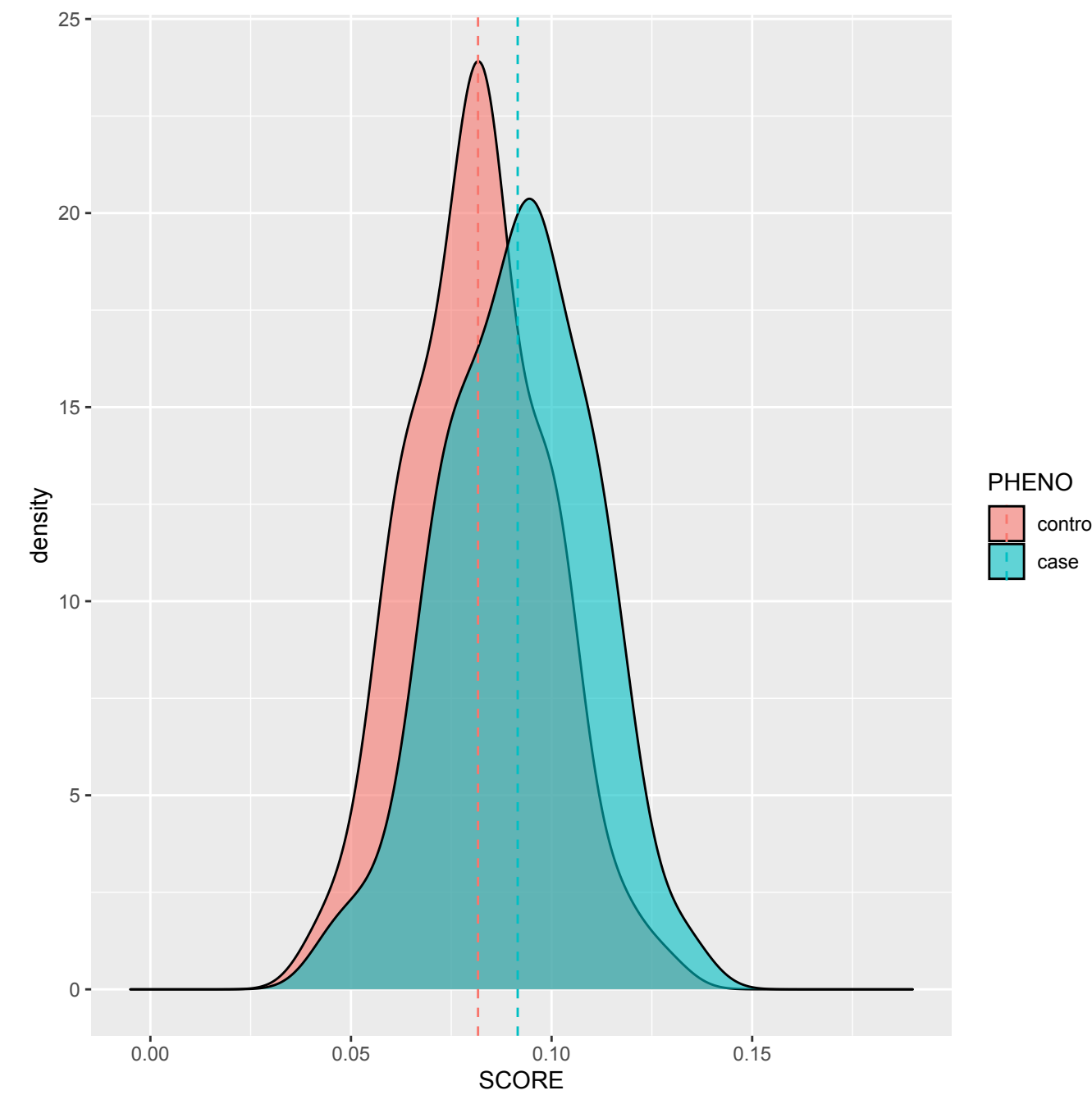

Supplement: Supplementary file 9 — Additional file 9: Figure S9. Density plots using 16 PD-associated SNPs from LPS data. Green color represents density for cases, and pink color represents for control. The x-axis represents polygenic risk score, and the y-axis represents density of samples. [file 40246_2021_357_MOESM9_ESM.pdf]

a

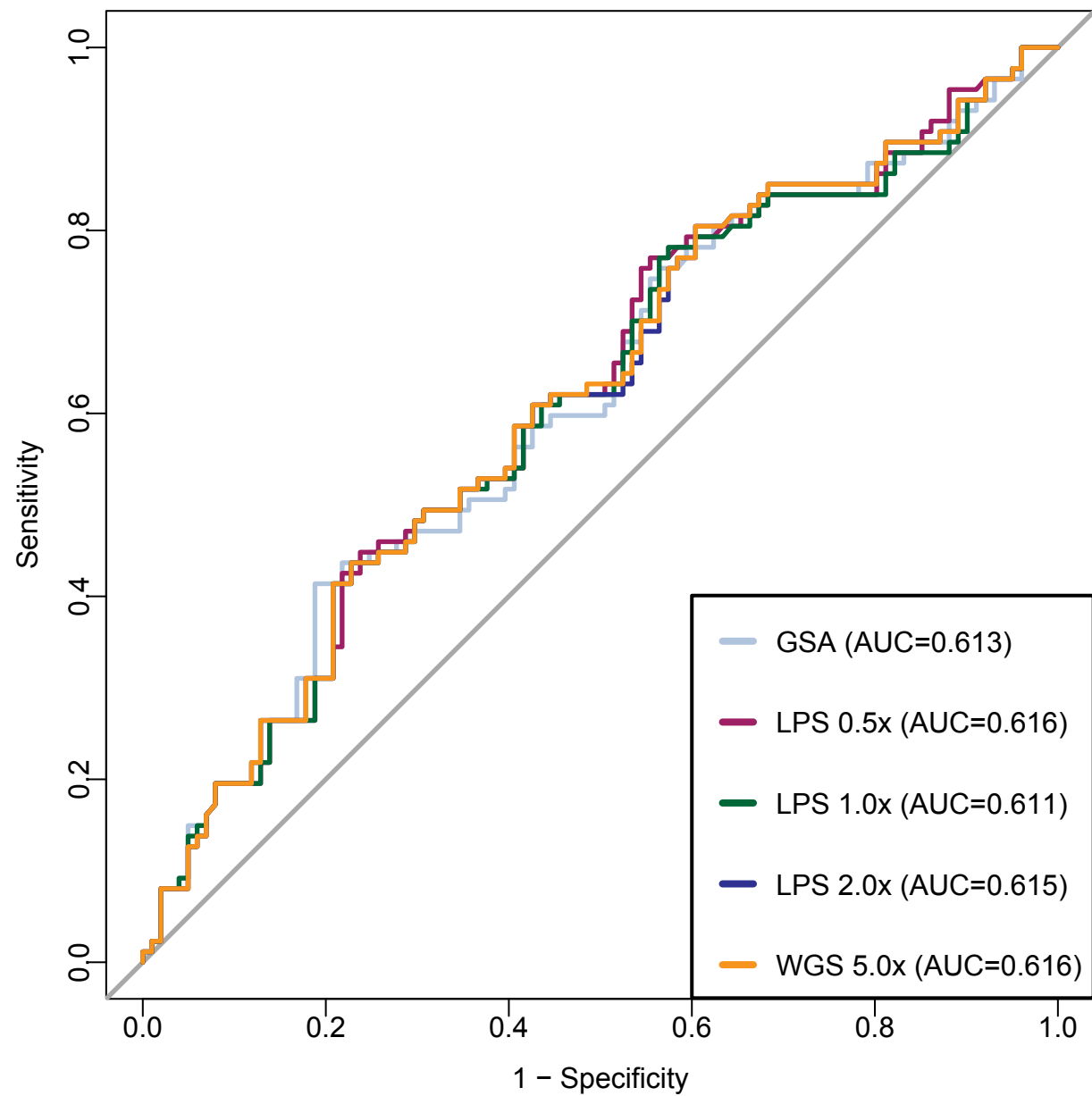

b

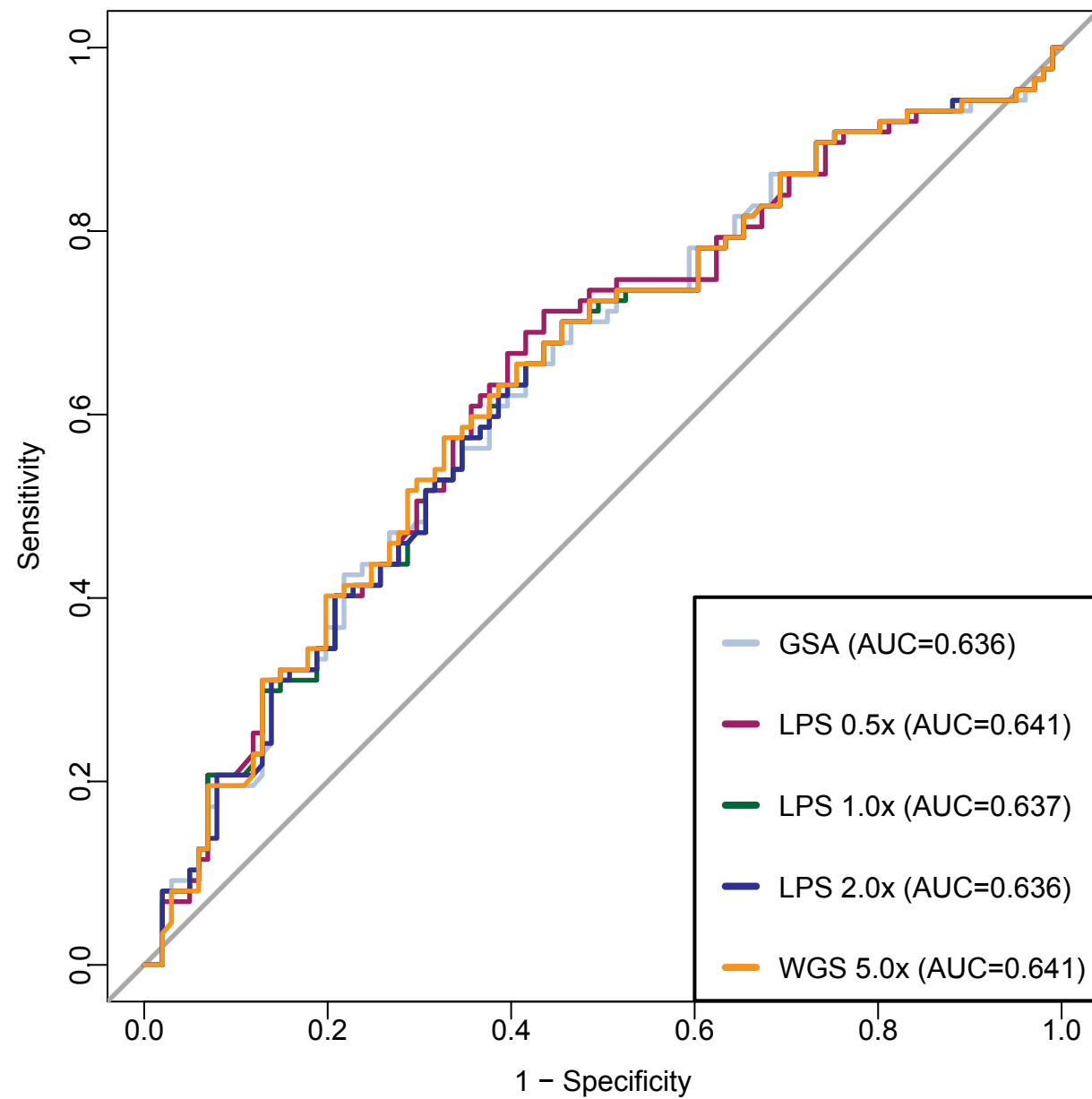

Supplement: Supplementary file 10 — Additional file 10: Figure S10. AUC of PRS analysis. Genotype imputation was conducted using the NARD reference panel by GLIMPSE. a PRS calculated based on 11 Asian SNPs, b PRS calculated based on nine European SNPs that were replicated in East Asian cohorts. [file 40246_2021_357_MOESM10_ESM.pdf]

a

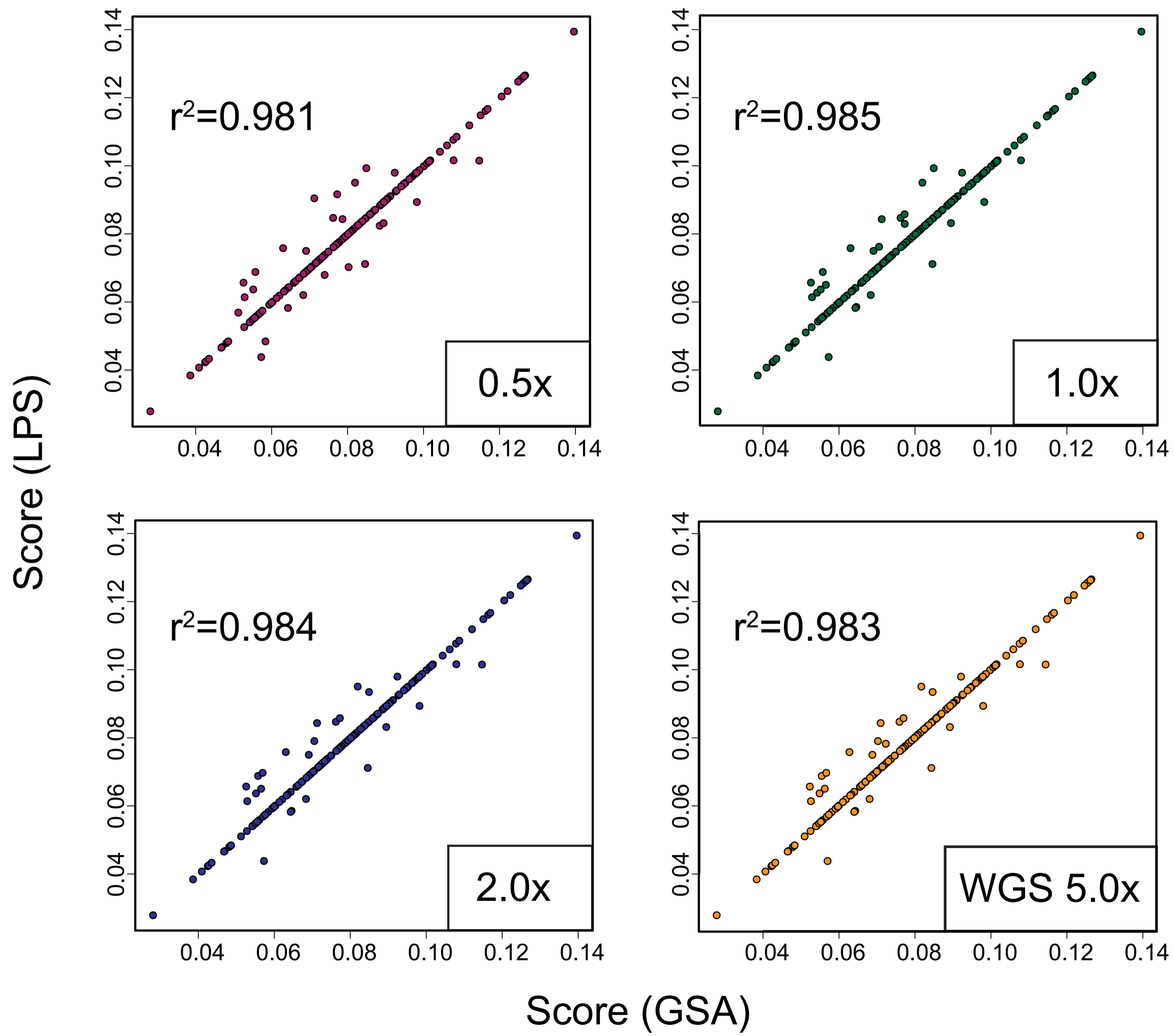

b

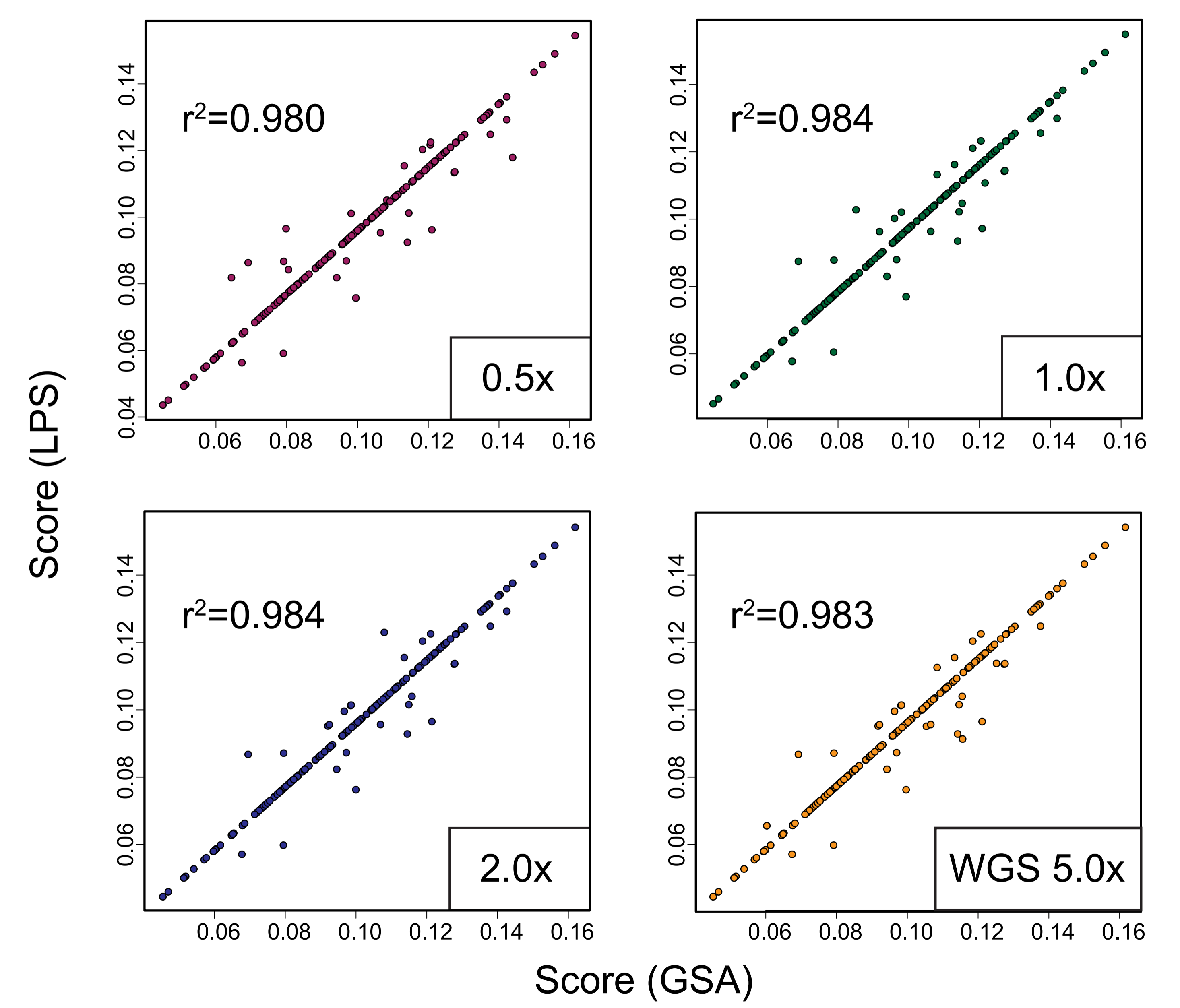

Supplement: Supplementary file 11 — Additional file 11: Figure S11. Correlation of PRS between GSA and LPS. a using 11 Asian SNPs, b using nine European SNPs that were replicated in East Asian cohorts. [file 40246_2021_357_MOESM11_ESM.pdf]
